# Ethanol-induced alterations of amino acids measured by *in vivo* microdialysis in rats: a meta-analysis

**DOI:** 10.1186/2193-9616-1-7

**Published:** 2013-05-17

**Authors:** Sarah Fliegel, Ines Brand, Rainer Spanagel, Hamid R Noori

**Affiliations:** Institute of Psychopharmacology, Central Institute of Mental Health, Faculty of Medicine Mannheim, University of Heidelberg, J5, 68159 Mannheim, Germany

**Keywords:** Microdialysis, Glutamate, GABA, Meta-analysis, Rat brain, Ethanol administration, Alcohol withdrawal

## Abstract

**Purpose:**

In recent years *in vivo* microdialysis has become an important method in research studies investigating the alterations of neurotransmitters in the extracellular fluid of the brain. Based on the major involvement of glutamate and γ-aminobutyric acid (GABA) in mediating a variety of alcohol effects in the mammalian brain, numerous microdialysis studies have focused on the dynamical behavior of these systems in response to alcohol.

**Methods:**

Here we performed multiple meta-analyses on published datasets from the rat brain: (i) we studied basal extracellular concentrations of glutamate and GABA in brain regions that belong to a neurocircuitry involved in neuropsychiatric diseases, especially in alcoholism (Noori et al., Addict Biol 17:827-864, 2012); (ii) we examined the effect of acute ethanol administration on glutamate and GABA levels within this network and (iii) we studied alcohol withdrawal-induced alterations in glutamate and GABA levels within this neurocircuitry.

**Results:**

For extraction of basal concentrations of these neurotransmitters, datasets of 6932 rats were analyzed and the absolute basal glutamate and GABA levels were estimated for 18 different brain sites. In response to different doses of acute ethanol administration, datasets of 529 rats were analyzed and a non-linear dose response (glutamate and GABA release) relationship was observed in several brain sites. Specifically, glutamate in the nucleus accumbens shows a decreasing logarithmic dose response curve. Finally, regression analysis of 11 published reports employing brain microdialysis experiments in 104 alcohol-dependent rats reveals very consistent augmented extracellular glutamate and GABA levels in various brain sites that correlate with the intensity of the withdrawal response were identified.

**Conclusions:**

In summary, our results provide standardized basal values for future experimental and *in silico* studies on neurotransmitter release in the rat brain and may be helpful to understand the effect of ethanol on neurotransmitter release. Furthermore, this study illustrates the benefit of meta-analyses using the generalization of a wide range of preclinical data.

## Background

*In vivo* microdialysis methods have been developed to study the quantity of the chemical composition of interstitial tissue fluids. This technique has been used to observe the extracellular neurotransmitter release in various brain regions of different species. Usually these studies first establish a baseline level of a specific neurotransmitter and subsequently investigate alterations in extracellular neurotransmitter concentrations in response to the administration of a certain drug or other manipulation.

Numerous microdialysis studies focus on amino acids, in particular glutamate and GABA, as these neurotransmitters are the key players in the excitatory and inhibitory network of the central nervous system (CNS) and are involved in a variety of neuropsychiatric diseases, including substance abuse and alcohol use disorders (Kalivas, 2009; Spanagel, 2009).

In recent years, the glutamate theory of alcoholism has emerged as a major theory in the addiction research field. In a seminal publication, David Lovinger and colleagues (Lovinger et al. 1989) demonstrated that N-methyl-D-aspartate (NMDA) receptor function was inhibited by ethanol. Further research using site-directed mutagenesis experiments identified putative binding sites for ethanol molecules at the NMDA receptor (for review, see Spanagel, 2009). Thus, the first level of interaction of alcohol with brain function concerns the NMDA receptor (but also the γ-aminobutyric acid A (GABA_A_; for an overview, see Vengeliene et al., 2008). The NMDA receptor is a ligand-gated ion channel with a heteromeric assembly of NR1, NR2 (A-D), and NR3 subunits, and genetic variants that affect the vulnerability to alcohol dependence within the genes encoding these subunits have been identified (Schumann et al., 2008; Domart et al., 2012; Tsai and Coyle, 2012). In addition to this direct interaction with the NMDA receptor, acute alcohol administration also affects glutamatergic neurons at the synaptic and cellular level and thereby releases glutamate. Although numerous microdialysis studies have examined the alcohol-induced glutamate release process, its concentration-dependency is less clear. It is further proposed that through various neuroadaptive responses that restore homeostasis, chronic alcohol consumption leads to an enhanced activity of the glutamatergic system in alcohol-dependent individuals (Tsai and Coyle, 1998; Spanagel and Kiefer, 2008; Ding et al., 2012). This glutamate-induced hyperexcitability within the CNS is uncovered during alcohol withdrawal. Acute alcohol withdrawal responses, which typically occur after discontinuation of prolonged and excessive alcohol ingestion, are associated with increased central glutamatergic transmission. Several studies employing brain microdialysis experiments in alcohol-dependent animals have shown augmented extracellular glutamate levels in various brain sites that correlate with the intensity of the withdrawal response (Rossetti and Carboni, 1995; Gass and Olive, 2008; Gass et al., 2011). This finding also translates into the human situation, as alcoholics undergoing acute withdrawal exhibit increased glutamate brain levels, as measured by magnetic resonance spectroscopy (Hermann et al., 2012).

As previously mentioned, other receptors or ion channels expressed within the CNS also have putative alcohol binding sites. In particular, the function of GABA_A_ receptors is enhanced by ethanol. The GABA_A_ receptor/chloride channel complex is a pentameric ligand-gated ion channel and the major inhibitory neurotransmitter receptor in the mammalian brain. Several subunits have been identified, with the majority of GABA_A_ receptors composed of α, β, γ and δ subunits (Barnard et al., 1998; Rewal et al., 2012). Using different receptor constructs, putative ethanol binding sites in the transmembrane domaines of the α/ β subunits of the GABA_A_ receptor have been identified (Mihic et al., 1997), and genetic variants within the genes encoding these subunits have been shown to affect the vulnerability to alcohol dependence (Cui et al., 2012; Frank et al., 2012; Uhart et al., 2012). Finally, some microdialysis studies have shown that acute alcohol also affects GABA release (Koob, 2004). Thus, consistent with the neuroadaptive changes that occur in the glutamatergic system, similar alterations might also occur in the GABAergic system following chronic alcohol administration.

Despite the important advantages of microdialysis measurements, the low spatiotemporal resolution remains a major drawback of these investigations. However, recent studies on the modeling of acute and chronic drug effects (Noori, 2012; Noori et al., 2012a) suggest that *in silico* analysis of the neurochemical processes provides complimentary information to overcome the experimental difficulties, particularly by enabling the observation of the dynamical multi-dimensional interactions of different transmitter systems with high spatiotemporal resolution. These computational methods rely on microdialysis results as initial setup parameters. Thus, comprehensive insights on the dynamical behavior of the extracellular concentrations of these neurochemical systems are of particular importance for understanding the neurobiology of alcohol abuse and alcoholism by conventional or *in silico* approaches. We have introduced a neurocircuitry (Noori et al., 2012a) that provides the foundation of such computational models. Using systematic data mining and clustering methods, we have identified specific brain regions and neurotransmitter systems, including glutamate and GABA, that are critical for understanding the spatiotemporal effects of drugs, especially alcohol, on the neurochemical mechanisms and processes in the rodent brain.

The main objective of the present study is to provide universally valid basal amino acid (glutamate and GABA) concentrations and their alterations due (i) to the administration of acute ethanol and (ii) during withdrawal, as measured by *in vivo* microdialysis experiments. Our previous studies (Frank et al., 2008; Noori et al., 2012b; Brand et al., 2013) suggest that meta regression analysis presents a suitable framework to approach this aim. Here, we use a similar strategy as in these studies and apply equivalent data mining and analytic methods.

Meta-analysis describes the integration of several primary studies using quantitative and statistical methods (Glass, 1976; Smith and Glass, 1977). The intention is to summarize the results of a large collection of individual studies in order to give a universally valid statement on specific topics. In particular, the effectiveness of a specific treatment or measure is investigated.

## Methods

### Data mining

A literature search was conducted on Pubmed (http://www.ncbi.nlm.nih.gov/pubmed/). No particular journal was preferred. The search included the specific brain region and the transmitter of interest as well as the keywords “rat” and “microdialysis”. Literature search for ethanol administration also included the keyword “ethanol” and “alcohol”. The selection criteria further included (i) rats of the age between 2 and 15 months and (ii) drug-naïve rats. Articles that did not comply with these criteria had to be excluded. Out of approximately 5000 publications, 245 publications fulfilled the selection criteria. In a second search we included “withdrawal”. Out of 43 publications, 11 publications fulfilled our stringent selection criteria for this additional meta-analysis.

The subsequent variables (i.-vii.) were obtained from the publications and used for further analysis:

i. Weight, age, gender and consciousness of the rats (if anaesthetics applied: agent and dose).
ii. Number of the animals used in each experiment.
iii. Absolute basal glutamate and GABA values. Different units were converted into molarity (nM).
iv. Sample time in min and perfusion rate in μl/min.
v. Peak % baseline (= highest divergence between maximum peak and baseline value) and peak time.
vi. Coordinates of probe placements according to the stereotaxic atlas of (Paxinos and Watson, 2007), Pellegrino et al. (Pellegrino and Cushman 1979), or König and Klippel (1974) as well as the shape, length and outer diameter of the probe membrane (mm), the calcium concentration and pH value of the Ringer solution or artificial CSF (mM), and the neurochemical detection assays.
vii. Doses of ethanol applied, as well as the route of administration (intravenous (i.v.) and intraperitoneal (i.p.) injections or local infusions).

### Statistical analysis

Usually a meta-analysis observes an entire experiment. Although we considered only selected values, we did not lose the relation to the experiment in total. The mean basal values are not collected from only one animal that means numbers, percentages etc. are associated to the whole experiment. We conducted the meta-analysis using fixed effect model (Hedges and Olkin, 1985), which utilizes the inverse of the number of animals of the studies as the weights to calculate a weighted average , where  represents the weighted average value as the weighted sum of the products of the mean values *x*_*i*_ from each experiment *i* (within a time interval of [0; 300] minutes) and the number of animals used in that particular study *n*_*i*_, and  denoting the total number of animals considered in the meta-analysis of the *k* studies. To guarantee the robustness of this model, we have analyzed the datasets statistically with respect to the experimental parameters by one-way analysis of variance (ANOVA) using the Holm-Bonferroni method with a global level of significance of α < 0.05 and identified significant heterogeneity factors.

One purpose of the analysis was to get the mean basal value of the two neurotransmitters glutamate and GABA measured in a defined brain region. According to our defined neurocircuitry for modelling acute and chronic effects of alcohol 19 brain regions were taken into consideration - from caudal to rostral: olfactory bulb (OB), prefrontal cortex (PFC), insula (Ins), nucleus accumbens (NAc), caudate putamen (CPu), septal region (S), bed nucleus of stria terminalis (BNST), globus pallidus (GP), hypothalamus (HyT), amygdala (Amy), habenula (Hb), hippocampus (Hc), thalamus (Th), subthalamic nucleus (STh), substantia Nigra (SN), ventral tegmental area (VTA), raphe nuclei (R), locus coeruleus (LC), and pons (Pn). As mentioned above, weighted values (concerning the number of rats, which were taken in one experiment) were used for calculation in order to get an average basal value. In addition to systematically examine those baseline values the second objective was the “peak % baseline” after acute administration of alcohol (i.p., i.v., s.c., local). A dose-dependent correlation analysis was conducted using the variables peak % baseline, peak time and the given dose of ethanol to determine the functional relationship between administered dose of ethanol and the alteration of glutamate and GABA concentrations, respectively. The third objective was the estimation of “peak % baseline” and “peak time” during alcohol withdrawal.

To analyse the data, one-way analysis of variance (ANOVA) using Holm-Bonferroni method with a global level of significance of α < 0.05 were performed. If any significance emerged, the respective weighted average basal value and standard error were calculated separately. Additionally forest plots were used to illustrate the influence of ethanol on the baseline values of glutamate in the prefrontal cortex and the nucleus accumbens. This graphical representation is a scattergram of the variables “experiment” and “average basal value” and “peak % baseline”, respectively.

## Results

### Baseline values for extracellular glutamate and GABA concentrations in different areas of the rat brain

**Figure 1:**
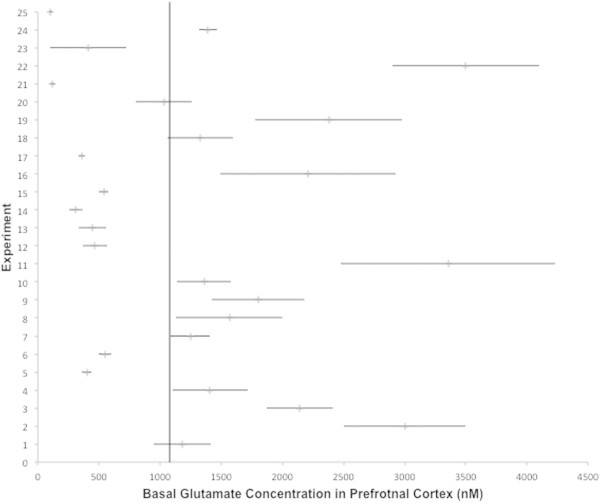
**Forest-plot of the basal glutamate values in the prefrontal cortex as measured in 24 experiments, ordered by year of publication.** Row 1 indicates the weighted average basal value and its standard error of mean (±SEM). The vertical line extends the weighted mean in order to compare the extracted data. 2 Hashimoto et al. (1995); 3 Stephans and Yamamoto (1995); 4 Robert et al. (1996); 5,6 Selim and Bradberry (1996); 7 Del Arco and Mora (1999); 8 Timmerman et al. (1999); 9 Del Arco and Mora (2000); 10 Del Arco and Mora (2002); 11 Pistis et al. (2002); 12 Harte and O'Connor (2004); 13 Giovannini et al. (2005); 14 Abekawa et al. (2006); 15 Calcagno et al. (2006); 16 Hugues et al. (2007); 17 Ballini et al. (2008); 18 Hernandez et al. (2008); 19 Huang et al. (2008); 20 Welty and Shoblock (2009); 21 Yamamura et al. (2009a); 22 Li et al. (2010a); 23 Lupinsky et al. (2010); 24 Carli et al. (2011); 25 Ohoyama et al. (2011).

**Figure 2:**
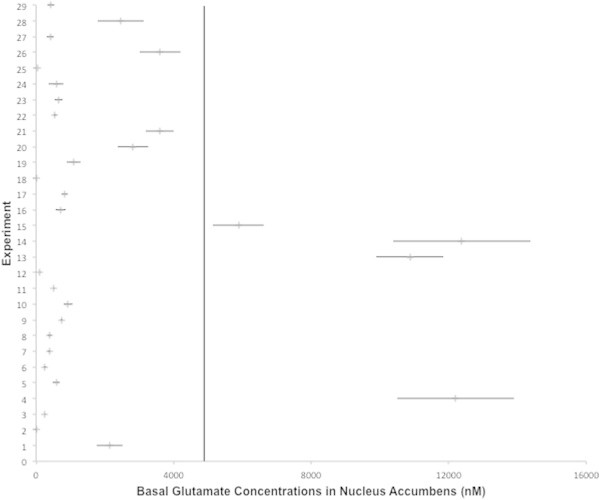
**Forest-plot of the basal value of glutamate in the nucleus accumbens as measured in 28 experiments, ordered by year of publication.** Row 1 indicates the weighted average basal value and its standard error of mean (±SEM). The vertical line extends the weighted mean in order to compare the extracted data. 2 Dahchour et al. (1994); 3 Selim and Bradberry (1996); 4 You et al. (1998); 5 Dalley et al. (1999); 6,7,8 Segovia et al. (1999); 9 Fu et al. (2000); 10 Quertemont et al. (2000); 11 Dawson et al. (2001); 12 Giorgetti et al. (2001); 13,14 Hemmati et al. (2001); 15 You et al. (2001); 16 Saulskaya and Mikhailova (2002); 17 Zangen and Hyodo (2002); 18 Hotsenpiller and Wolf (2003); 19 Mikhailova (2003); 20 Xi et al. (2003a); 21 Quarta et al. (2004); 22 Saulskaya and Soloviova (2004); 23 Shou et al. (2004); 24 Saul'skaya and Mikhailova (2005); 25 Ito et al. (2006); 26 Lallemand et al. (2006); 27 Hernandez (2008); 28 Huang et al. (2008); 29 Li et al. (2010b).

Literature search revealed 245 publications that fulfilled the selection criteria for baseline values of glutamate and GABA. Out of these 43.3% were published before the year 2000, 51.8% between 2000 and 2010 and 4.5% after 2010. Altogether 6932 animals were used in these experiments. Average basal values, as well as the statistical distribution (i.e., median, maximum and minimum) are represented in Table 1 (glutamate) and Table 2 (GABA) for 18 different brain regions respectively (for the habenula no data could be retrieved from Pubmed). The forest plots (Figures 1 and 2) represent the basal values of glutamate in the PFC and the NAc, respectively. Rapid microelectrode measurements of glutamate in the PFC (Hascup et al., 2010), glutamate measurements with oxidase-coated biosensors in the AMY and NAc (Gass et al., 2011) as well as a variety of control experiments (Timmerman and Westerink, 1997; Sun et al., 2011) suggest the neuronal origin of these concentrations.

**Table 1:** **Average basal values (nM) of glutamate in awake animals as well as the statistical distribution of the data (i.e., median, maximum and minimum)**

Brain region	Glutamate: average basal value ± sEM [nM]	Median	Max	Min
(Number of rats)				
**Olfactory Bulb** (30)	3857 ± 2057	4681	3307	6055
**Prefrontal Cortex** (445)	1182 ± 236	1290	3500	105
**Insular Cortex** (6)	1750 ± 320	-	-	-
**Nucleus Accumbens** (661)	2135 ± 382	623	12379	10
**Caudate Putamen** (675)	1009 ± 166	735	8100	25
**Bed Nucleus of Stria Terminalis** (7)	830 ± 70	-	-	-
**Globus Pallidus**	435 ± 153	400	673	171
Sprague–Dawley (42)				
Wistar (39)	876 ± 381	1518	1905	236
**Hypothalamus** (63)	1178 ± 373	492	3500	24
**Amygdala** (138)	4475 ± 1779	835	10980	32
**Hippocampus** (301)	2616 ± 513	1480	18940	50
**Thalamus** (71)	842 ± 280	705	1640	114
**Subthalamic Nucleus** (30)	118 ± 1	-	-	-
**Substantia Nigra** Sprague–Dawley (487)	136 ± 41	115	518	88
Wistar (75)	517 ± 210	500	684	110
**Ventral Tegmental Area**	205 ± 68	177	410	114
Sprague–Dawley (184)				
Wistar (17)	571 ± 342	504	733	275
Long-Evans (59)	1294 ± 654	1295	1489	1100
**Raphe** (7)	1243 ± 92	-	-	-
**Locus Coeruleus** (100)	2430 ± 730	4400	10750	58
**Pons** (26)	75 ± 5	-	-	-

**OB**: Guevara-Guzman, R., et al. (2000) **PFC**: Abekawa et al. (2006); Ballini et al. (2008); Calcagno et al. (2006); Carli et al. (2011); Del Arco and Mora (1999); Del Arco and Mora (2000); Del Arco and Mora (2002); Giovannini et al. (2005); Harte and O'Connor (2004); Hashimoto et al. (1995); Hernandez et al. (2008); Huang et al. (2008); Hugues et al. (2007); Li et al. (2010b); Lupinsky et al. (2010); Ohoyama et al. (2011); Pistis et al. (2002); Qi et al. (2012); Robert et al. (1996); Selim and Bradberry (1996); Stephans and Yamamoto (1995); Timmerman et al. (1999); Welty and Shoblock (2009); Yamamura et al. (2009a) **Ins:** Guzman-Ramos et al. (2010) **NAc**: Dahchour et al. (1996); Dalley et al. (1999); Dawson et al. (2001); Ericson et al., (2011); Fu et al. (2000); Giorgetti et al. (2001); Hemmati et al. (2001); Hernandez et al. (2008); Hotsenpiller and Wolf (2003); Huang et al. (2008); Ito. et al. (2006); Lallemand et al. (2006); Li et al.(2010a); Mikhailova (2003); Quarta et al. (2004); Quertemont et al.(2000); Saulskaya and Mikhailova (2002); Saul'skaya and Mikhailova (2005); Saulskaya and Soloviova (2004); Segovia et al. (1999); Selim and Bradberry (1996); Shou et al. (2004); Xi et al. (2003b); You et al. (2001); You et al. (1998); Zangen and Hyodo (2002) **CPu:** Anderson and DiMicco (1992); Battaglia et al. (1997); Bert et al. (2002); Carboni et al. (1993); Dawson et al. (2001); Dawson et al. (2003); Del Arco et al. (1998); Fantin et al. (2007); Ferraro et al. (1998); Hashimoto et al. (1995); Hernandez et al. (2008); Lillrank et al. (1994); Mark et al. (2004); Massieu et al. (1995); Meeusen et al. (1997); Melani et al. (2003); Molchanova et al. (2004a); Molchanova, et al. (2004b); Morales-Villagran and Tapia (1996); Morari et al. (1996); Morari et al. (1993); Morari (1994); Northrop et al. (2011); Parrot et al. (2003); Segovia et al. (1997); Segovia et al. (1999); Segovia et al. (2001); Stephans and Yamamoto (1995); Takeda et al. (2003); Toth et al. (1993); Yamada et al. (2009); Yamamoto et al. (1999) **BST**: Forray et al. (1999) **GP**: Biggs et al. (1997a); Biggs and Starr (1997b); Chapman and See (1996); Fantin et al. (2007); Ferraro et al. (1998); Galeffi et al. (2003); Kretschmer (2000); Li et al. (2010a); Sizemore et al. (2000); Windels et al. (2000); Windels et al. (2005) **HyT**: Anderson and DiMicco (1992); Azuma et al. (1996); Ferraro et al. (1999); Keck et al. (2000); Mason et al. (1997); Melis et al. (2004); Succu et al. (2006) **Amy**: Kaura et al. (1995); Mucignat-Caretta et al. (2006); Quertemont et al. (1999); Quertemont et al. (1998); Roberto et al. (2004b); Skorzewska et al. (2009) **Hc**: Ballini et al. (2008); Biggs et al. (1992); Clinckers et al. (2005); Dawson et al. (2001); Ferraro et al. (1997a); Giovannini et al. (2005); Giovannini et al. (2001); Giovannini et al. (1998); Hossain et al. (2008); Katoh et al. (1997); Kuntz et al. (2004); Langlais and Zhang (1993); Oreiro-Garcia et al. (2007); Rakovska et al. (1998); Rosi et al. (2004); Rowley et al. (1995); Shimizu et al. (1998); Takeda et al. (2004); Takeda et al. (2002); Tanaka et al. (2004); Ueda and Tsuru (1995); Wislowska-Stanek et al. (2008); Zhu et al. (2008); Zuiderwijk et al. (1996) **Th**: Abarca et al. (2000); Banerjee and Snead (1995); Ferraro et al. (1997b); Hazell et al. (1993); Langlais and Zhang (1993); Nyitrai et al. (1999); Terzioglu et al. (2006) **STh**: Ampe et al. (2007) **SN:** Bianchi et al. (1998); Biggs et al. (1995); Boulet et al. (2006); Fantin et al. (2007); Ferraro et al. (1998); Ferraro et al. (2001); Galeffi et al. (2003); Hatzipetros and Yamamoto (2006); Marti et al. (2002); Morari et al. (1998); Nyitrai et al. (1999); Robelet et al. (2004); Rosales et al. (1997); Yamamura et al. (2009ba) **VTA:** Frantz et al. (2002); Fu et al. (2000); Harte and O'Connor (2004) Harte and O'Connor (2005); Kretschmer et al. (2000); O'Dell (2004); Pehek et al. (2006); Timmerman et al. (1999); Wang et al. (2005); Wolf and Xue (1998); Wolf and Xue (1999); You et al. (2007) **R**: Varga et al. (1998) **LC:** Feng et al. (1997); Feng et al. (1995); Hoshi et al. (1996); Hoshi et al. (1997); Liu et al. (1999); Singewald et al. (1995); Sullivan et al. (2000); Timmerman, et al. (1999); Tokuyama et al. (1998); Zhang et al. (1994) **Pn:** Sato et al. (2007).

**Table 2:** **Average basal values (nM) of GABA in awake animals as well as the statistical distribution of the data (i.e. median, maximum and minimum)**

Brain region	GABA: average basal	Median	Max	Min
(Number of rats)	value ± SEM [nM]			
**Olfactory Bulb** (30)	73 ± 46	61	80	43
**Prefrontal Cortex**	34 ± 12	32	50	25
Sprague–Dawley (131)				
Wistar (80)	89 ± 33	118	170	10
**Nucleus Accumbens** (167)	90 ± 22	33	764	13
**Caudate Putamen**	17 ± 5	19	130	6
Sprague–Dawley (341)				
Wistar (300)	78 ± 22	110	660	1
**Septal Region** (17)	640 ± 420	488	775	200
**Bed Nucleus of Stria Terminalis** (7)	110 ± 20	-	-	-
**Globus Pallidus** (198)	21 ± 6	19	83	7
**Hypothalamus** (56)	29 ± 10	17	92	5
**Amygdala** (128)	56 ± 20	16	830	2
**Hippocampus** (302)	97 ± 19	95	2500	1
**Thalamus** (100)	228 ± 70	60	870	8
**Subthalamic Nucleus** (33)	9 ± 5	9	9	9
**Substantia Nigra** (454)	18 ± 4	15	145	4
**Ventral Tegmental Area** (202)	16 ± 6	23	43	8
**Locus Coeruleus** (6)	6 ± 1	-	-	-
**Pons** (26)	90 ± 7	-	-	-

**OB**: Guevara -Guzman et al. Guevara-Guzman et al. (2000) **PFC**: Ballini et al. (2008); Del Arco and Mora (1999); Del Arco and Mora (2000); Del Arco and Mora (2002); Grobin and Deutch (1998); Harte and O'Connor (2004); Hernandez et al. (2008); Huang et al.(2008); Ohoyama et al. (2011); Petkova-Kirova et al. (2008) Pistis et al. (2002); Welty and Shoblock (2009); Yamamura et al. (2009a) **NAc:** Dahchour (1996); Ferraro et al. (1996a); Hazell (1993); Hemmati et al. (2001); Hernandez (2008); Huang et al. (2008); Lindefors et al. (1992); Reynolds et al. (1999); Segovia (1999); Shou et al. (2004); Smith and Sharp (1994); Tanganelli et al. (1994); Xi et al. (2003a) **CPu**: Anderson and DiMicco (1992); Bourdelais and Deutch (1994); Del Arco et al. (1998); Fantin et al. (2007); Ferraro et al. (1998); Ferraro et al. (1997b); Hernandez et al. (2003 ); Hernandez et al. (2008); Hondo et al. (1995); Lillrank et al. (1994); Meeusen et al. (1997); Melani et al. (2003); Molchanova et al. (2004a); Morari et al. (1996); Morari et al. (1993); Morari et al. (1994); Segovia et al. (1997); Segovia et al. (1999); Semba et al. (1995); Takeda et al. (2003); Wang et al. (2007); Yamamoto et al. (1999) **S**: Giovannini et al. (1994); Sotomayor-Zarate et al. (2010) **BST**: Forray et al. (1999) **GP**: Chapman and See, (1996) Cowen et al. (1998); Fantin et al. (2007); Ferraro et al. (1998); Ferraro et al. (1997a); Ferraro et al. (2000); Galeffi et al. (2003); Inui et al. (2009); Littlewood et al. (2006); O'Connor etal. (1998); Rimondini et al. (1994); Rimondini et al. (1996); Sizemore et al. (2000); Sommer et al. (1996); Windels et al. (2000); Windels et al. (2005) **Hy**t: Anderson and DiMicco (1992); Dong et al. (2006); Ferraro et al. (1999); Ferraro et al. (1996b); Katoh et al. (1997); Voisin et al. (1994) **Amy**: Kaura et al. (1995); Mucignat-Caretta et al. (2006); Quertemont et al. (1999); Rea et al. (2009); Roberto et al. (2010); Roberto et al. (2004b); Skorzewska et al. (20099) **Hc**: Ballini et al. (2008); Biggs et al. (1992); Dalby (2000); de Groote and Linthorst (2007); Ferraro et al. (1997b); Giovannini et al. (2001); Giovannini et al. (1998); Hossain et al. (2008); Katoh et al. (1997); Kuntz et al. (2004); Langlais and Zhang (1993); Oreiro-Garcia et al. (2007); Rakovska et al. (1998); Rosi et al. (2004); Rowley et al. (1995); Takeda et al. (2004); Takeda et al. (2002); Ueda and Tsuru (1995); Wislowska-Stanek et al. (2008); Yoshida et al. (2007); Zuiderwijk et al. (1996) **Th**: Banerjee and Snead (1995); Dalby (2000); Ferraro et al. (1996b); Ferraro et al. (2001); Juhasz et al. (1997); Langlais and Zhang (1993); Mark (2004); Nyitrai et al. (1999); Terzioglu et al. (2006) **STh**: Ampe et al. (2007); Yamamura et al. (2009a) **SN**: Bianchi et al. (1998); Biggs et al. (1995); Boulet et al. (2006); Bustamante et al. (2002); Fantin et al. (2007); Ferraro et al. (1998); Ferraro et al. (2001); Galeffi et al. (2003); Herrera-Marschitz et al. (1996); Invernizzi et al. (2007); Mark et al. (2004); Matuszewich and Yamamoto (1999); Morari et al. (1996); Ochi et al. (2004); Rosales et al. (1997); Sayin et al. (1995); Sommer et al. (1996); Windels et al. (2000); Windels et al. (2005); You et al. (1996a); You et al. (1996b); You et al. (2007) **VTA**: Bankson and Yamamoto (2004); Frantz et al. (2002); Harte and O'Connor (2004); O'Dell and Parsons (2004); Winter et al. (2008); Yan et al. (2005); You et al. (2007) **LC**: Singewald et al. (1995) **Pn**: Sato et al. (2007).

Numerous experimental variables are known to have an impact on the relative recovery of an analyte and thereby influence the concentration per sample and the baseline values measured. Most critical parameters are the flow rate of the perfusate, probe size, the composition of the perfusate - particularly the Ca^2+^ concentration, and the analytical technique for determining the neurotransmitter concentrations. The statistical distribution of these parameters within our datasets (Table 3) suggests a dense distribution of the parameters around their averages and a lack of significant heterogeneity in the applied ranges. ANOVA performed on the weighted averages with respect to these parameters reflected this absence of variance and suggests the robustness of our analysis in agreement with previous studies (Frank et al., 2008; Noori et al., 2012b; Brand et al., 2013). This result is not in contrast to the previous experimental observation but underlines the awareness of the study designers of the importance of these parameters. This was particularly reflected in the choice of the shape of the probes (99% I-shaped) and the transmitter detection systems. Almost all studies (98%) used high performance liquid chromatography (HPLC) and fluorecence detection systems for glutamate quantifications, whereas the vast majority of the studies measuring GABA utilize HPLC and coulometric electrochemical detection assays. However, it should also be mentioned that the majority of the studies used in the present study did not report the time point of measurement with respect to circadian rhythms. Recent studies (Castaneda et al., 2004; Hampp et al., 2008) suggest that the neurotransmitter levels measured by *in vivo* microdialysis are under the control of the circadian clock and vary with the time of the day. The lack of information on this issue in most of the publications might have a non-negligible impact on our analysis.

**Table 3:** **Statistical distribution of the microdialysis procedure parameters within the meta-analyzed datasets**

	Average	Median	Max	Min
**Flow Rate (μl/min)**	1.7	2.0	4.0	0.5
**Ca** ^**2+**^ **(mM)**				
aCSF (53%)	1.2	1.2	2.5	0.57
Ringer Solution (30%)	1.9	2.2	3.4	1.0
Krebs-Ringer-Phosphate Solution (9%)	1.5	1.2	3.4	1.0
Modified Ringer Solution (7%)	1.4	1.2	2.3	1.0
Dulbecco Phosphate Buffer Saline (1%)	1.2	1.2	1.2	1.2
**pH-value (Perfusate)**	7.4	7.4	7.4	6.0
**Probe Size**				
Length (mm)	2.3	2.0	5.0	1.0
Outer Diameter (mm)	0.3	0.3	0.6	0.15

The compliance of the average values and the median in the flow rates and in the different calcium concentrations within the composition of perfusates suggest a lack of heterogeneity and a high level of standardization in the general experimental design of microdialysis measurements.

Most of the experiments used Sprague–Dawley (43.4%) and Wistar rats (42.3%). A smaller percentage used Lister-Hooded (2.4%) and Long-Evans (3.1%) rats. Statistical analysis shows statistically significant differences of average basal values of rat strain in several brain regions. Most of them occurred between Wistar and Sprague–Dawley rats (strain differences shown in Table 1 and 2). In particular, GABA levels in the PFC and CPu were significantly different between Sprague–Dawley and Wistar rats (F_1,6_ = 6.03; resp. F_1,10_ = 4.76; P < 0.05). Furthermore, glutamate levels showed a statistically significant difference between Sprague–Dawley and Wistar rats in the GP (F_1,5_ = 9.11; p < 0.05), the SN (F_1,9_ = 4.67; P < 0.05), and the VTA (F_2,8_ = 4.26; P < 0.05). In general, the average basal values seem not to depend on gender. However, with the exception of measurements in the OB, which were performed only on female animals (n = 100), the majority of the remaining studies (96.5%) used male rats. Hence, statistical analysis did not reveal any gender-specific significant differences, but due to the low number of female rats it is difficult to draw any certain conclusion. In order to minimize age-related variations, only values obtained from adult animals (between 2 and 10 months of age) were considered for the analysis. The weight of the animals was Gaussian normal distributed around 300 g. The dominant part of the experiments (78%) was conducted on awake, conscious and freely moving animals. In the remaining studies, animals were maintained under anaesthesia during the experiment, which often induced statistically significant effects on the basal neurotransmitter concentrations (Table 4). Previous studies (Lillrank et al., 1994; Rozza et al., 2000; Dong et al., 2006; Westphalen and Hemmings, 2006) already suggest a significant impact of the anaesthetics on the forebrain glutamate and GABA levels. Our analysis further supports the suggestion that the application of different anaesthetics such as halothane, urethane and pentobarbital increase the level of glutamate significantly in Th (F_1,6_ = 80.12; P < 0.05), SN (F_1,14_ = 6.3; P < 0.05), and VTA (F_1,10_ = 83.53; P < 0.05). In addition, chloral hydrate appeared to also have enhancing effects on the GABA release in the SN (F_1,4_ = 216.28; P < 0.05) (Table 4).

**Table 4:** **Significantly different average basal values (nM) of glutamate and GABA (in comparison to Tables** 1 **and** 2 **) in anesthetized rats**

Brain region/ transmitter (number of animals)	Average basal value ± SEM	Median	Max	Min
**Thalamus/Glu (8)**	6600 ± 300	-	-	-
**Substantia Nigra/Glu (16)**	684 ± 259	699	863	440
**Ventral Tegmental Area/Glu (12)**	4607 ± 392	-	-	-
**Ventral Tegmental Area/GABA (6)**	226 ±79	-	-	-

**Glu-Th**: Juhasz et al. (1997) **Glu-SN**: Bustamante et al. (2002); Herrera-Marschitz et al. (1996); Windels et al. (2000); Windels et al. (2005); You, et al. (1996a); You et al. (1996b) **Glu-VTA:** You et al. (2001) **GABA-VTA**: Winter et al. (2008).

### Alcohol-induced glutamate and GABA release in different areas of the rat brain

**Figure 3:**
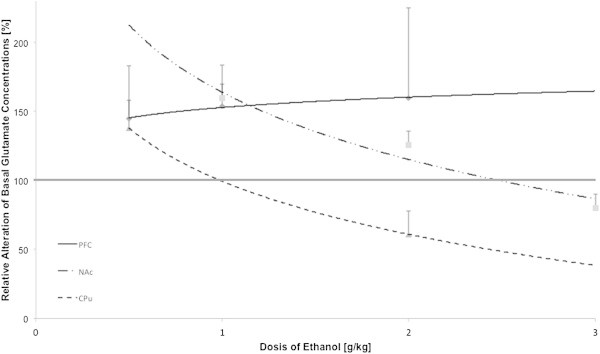
**Dose-dependent ethanol induced changes of extracellular glutamate concentrations (nM) in the prefrontal cortex (PFC), nucleus accumbens (NAc) and caudate putamen (CPu) of rats.**

Our literature search revealed 17 publications that were in agreement with our selection criteria for acute ethanol exposure. Out of these, 66 values were extracted. Altogether 529 animals were used in the experiments. Observation of seven brain regions fulfilled the selection criteria: AMY, GP, HC, NAc, PFC, CPu, and VTA. In general, alcohol was administered via three routes: (i) almost 90% of the experiments used intraperitoneal (i.p.) injections in a dose between 0.5 and 3.0 g/kg body weight; (ii) local infusion (100–1000 mM) of alcohol in 8% of the studies; and (iii) the remaining experiments applied ethanol orally (20% ethanol). The average magnitude of increase/decrease comparing to the baseline concentrations (peak % baseline) and the average peak time are presented in the Tables 5 and 6. The correlation analysis shows a non-uniform (region-dependent) interaction between ethanol and the release of glutamate and GABA. In particular, ethanol-induced alterations in glutamate concentrations appear to depend on the network properties such as the connectivity of the brain regions within the neurocircuitry for modelling drug effects. This observation is best reflected in the analysis of the PFC, NAc and CPu (Figure 3). While ethanol increases the glutamate concentrations in the PFC in a dose-dependent fashion, it simultaneously decreases the extracellular levels of glutamate in the NAc and CPu. In contrast GABA concentrations were elevated in the NAc following the same doses of alcohol.

**Table 5:** **Average ethanol-induced alterations of glutamate and GABA as measured by** ***in vivo*** **microdialysis experiments**

EtOH dosis (g/kg)	0.5	1.0	2.0	3.0
Brain region/transmitter (number of animals)	Peak % baseline (Peak time [min])			
**Prefrontal Cortex/Glu (44)**	145 (40)	154 (57)	160 (20)	
**Nucleus Accumbens/Glu (186)**		160 (53)	126 (49)	80 (80)
**Nucleus Accumbens/GABA (82)**		135 (58)	97 (65)	73 (90)
**Caudate Putamen/Glu (11)**	138 (NN)		61 (20)	

**Glu-PFC**: Selim and Bradberry (1996); **Glu-NAc**: Dahchour et al. (1994); Dahchour et al. (1996); Kashkin and De Witte (2004); Selim and Bradberry (1996); Yan et al. (1998) **GABA-NAc:** Dahchour et al. (1994); Dahchour et al. (1996); **Glu-CPu:** Carboni et al. (1993); Smith et al. (2004).

**Table 6:** Local infusion of ethanol in the AMY enhances GABA levels significantly, while glutamate release remains almost unchanged (Glu: Roberto et al. (2004b) GABA: Roberto et al. (2004a)

EtOH dosis (mM)	100	300	1000
		Peak % Baseline	
**Amygdala/Glu**	110	104	113
**Amygdala/GABA**	127	-	182

### Alcohol withdrawal-induced glutamate and GABA release in different areas of the rat brain

**Figure 4:**
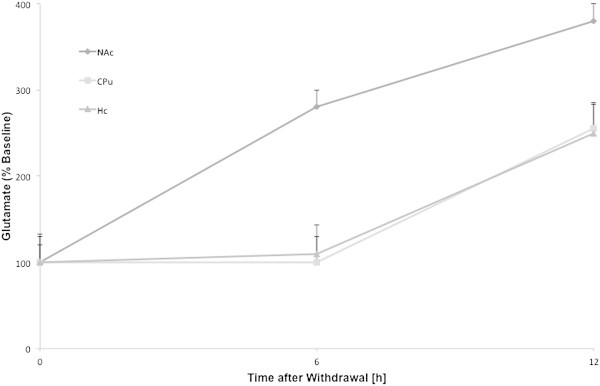
**The time course of withdrawal induced enhancements in the glutamate levels relative to the respective basal values in nucleus accumbens, caudate putamen and hippocampus.** The time course was not provided for central amygdala.

On the basis of our selection criteria for ethanol withdrawal, 11 articles (n = 104 rats) were extracted. All studies used freely moving male rats with a strain distribution of 55% Wistar and 45% Sprague Dawley animals. The experiments measured the amino acids alterations in an interval of [2; 12] hours after last exposure to alcohol within different brain regions (Table 7 and Figure 4) with significant enhancements of extracellular glutamate and GABA levels due to acute ethanol withdrawal.

**Table 7:** **The effects of acute ethanol withdrawal on extracellular amino acid concentrations in rats**

Brain Region	Glutamate	GABA
(Number of rats)		
**Central Amygdala (21)**	216%	360%
**Nucleus Accumbens (39)**	370%	
**Caudate Putamen (13)**	255%	
**Hippocampus (31)**	240%	100%

**Glu-Central AMY:**, 2004b; **GABA-Central AMY:**, 2004a Roberto et al. 2010; **Glu-NAc**, 1998; Dahchour and De Witte 1999b 2000 2000; Melendez et al., 2005; Saellstroem Baum et al. 2006; **Glu-CPu:**, 1995; **Glu-HC:**, 1999a 2003; **GABA-HC:**, 1999a 2003

## Discussion

To investigate the effects of a specific drug on amino acid release in the rat brain, *in vivo* microdialysis is an ideal method. Nevertheless, experimental parameters should be defined more precisely, as they can largely vary between different publications; however, there are no universal instructions concerning the number of animals, gender, age, doses of applied drugs, state of consciousness and weight in these studies. Our meta-analysis shows general robustness of the observations for glutamate and GABA release with respect to experimental parameters such as gender and state of consciousness of the animals, and provides universal references for the basal concentrations of glutamate and GABA in a number of brain regions. However, the observed statistical differences of glutamate and GABA neurotransmission in specific brain regions as a consequence of the administration of anaesthetics and strain of the animals suggest particular cautiousness in establishing baseline measurements with respect to these variables.

Our analysis further reflects the highly complex mechanisms underlying the actions of ethanol on the release properties of amino acids. While different doses of ethanol enhance the basal levels of glutamate in the PFC (Table 4 and Figure 3), the magnitude of the alterations appear to be nonlinearly dependent on the applied doses. In addition, the negative correlation of the administered doses of ethanol and the changes in amino acid concentrations in the dorsal and ventral striatum suggest the involvement of feedback mechanisms and the activation of additional secondary regulatory processes in the subcortical brain structures by alcohol (Noori et al., 2012a).

In general, the multi-scale involvement of glutamate and GABA in information processing in the brain (from synaptic to network interactions) and the interactions between these transmitters make it difficult to identify the key components of the ethanol-induced alterations. In light of these difficulties, *in silico* experiments might represent an alternative strategy to capture the dynamical complexity of these interactions and provide further neurobiological insights on the relevant processes that are not measurable simultaneously in real-world experiments.

## Conclusion

In conclusion, this meta-analysis approach may be helpful for the optimal systematic design of future *in vivo* microdialysis and *in silico* experiments on neurotransmitter release and ethanol-related processes, to therefore attain a better comparability between those studies. Furthermore, the basal extracellular concentrations of glutamate and GABA in 18 different brain sites, as well as the quantitative and qualitative measures for the acute action of ethanol on these neurotransmitters provide the necessary setup parameters for *in silico* studies.

### Limitations

Despite the numerous advantages of meta-analysis approaches, their main problem remains the lack of essential information in the publications. Many potentially important articles had to be excluded from our analysis because crucial information was missing, such as the number of animals used or standard errors of the mean. In addition, it should be noted that in the majority of studies, circadian rhythmicity was not considered and thus the time point of the measurement was oftentimes excluded. Recent studies (Casteneda et al., 2004; Hampp et al., 2008) indicate that there is a relationship between the concentrations of neurotransmitters, as measured by *in vivo* microdialysis, and the time of measurement (day/night).

## References

[CR1] Abarca C, Silva E, Sepulveda MJ, Oliva P, Contreras E (2000). Neurochemical changes after morphine, dizocilpine or riluzole in the ventral posterolateral thalamic nuclei of rats with hyperalgesia. Eur J Pharmacol.

[CR2] Abekawa T, Ito K, Koyama T (2006). Role of the simultaneous enhancement of NMDA and dopamine D1 receptor-mediated neurotransmission in the effects of clozapine on phencyclidine-induced acute increases in glutamate levels in the rat medial prefrontal cortex. Naunyn Schmiedebergs Arch Pharmacol.

[CR3] Ampe B, Massie A, D'Haens J, Ebinger G, Michotte Y, Sarre S (2007). NMDA-mediated release of glutamate and GABA in the subthalamic nucleus is mediated by dopamine: an in vivo microdialysis study in rats. J Neurochem.

[CR4] Anderson JJ, DiMicco JA (1992). The use of microdialysis for studying the regional effects of pharmacological manipulation on extracellular levels of amino acids–some methodological aspects. Life Sci.

[CR5] Azuma S, Kodama T, Honda K, Inoue S (1996). State-dependent changes of extracellular glutamate in the medial preoptic area in freely behaving rats. Neurosci Lett.

[CR6] Ballini C, Corte LD, Pazzagli M, Colivicchi MA, Pepeu G, Tipton KF, Giovannini MG (2008). Extracellular levels of brain aspartate, glutamate and GABA during an inhibitory avoidance response in the rat. J Neurochem.

[CR7] Banerjee PK, Snead OC (1995). Presynaptic gamma-hydroxybutyric acid (GHB) and gamma-aminobutyric acidB (GABAB) receptor-mediated release of GABA and glutamate (GLU) in rat thalamic ventrobasal nucleus (VB): a possible mechanism for the generation of absence-like seizures induced by GHB. J Pharmacol Exp Ther.

[CR8] Bankson MG, Yamamoto BK (2004). Serotonin-GABA interactions modulate MDMA-induced mesolimbic dopamine release. J Neurochem.

[CR9] Barnard EA, Skolnick P, Olsen RW, Mohler H, Sieghart W, Biggio G, Braestrup C, Bateson AN, Langer SZ (1998). International Union of Pharmacology. XV. Subtypes of gamma-aminobutyric acidA receptors: classification on the basis of subunit structure and receptor function. Pharmacol Rev.

[CR10] Battaglia G, Monn JA, Schoepp DD (1997). In vivo inhibition of veratridine-evoked release of striatal excitatory amino acids by the group II metabotropic glutamate receptor agonist LY354740 in rats. Neurosci Lett.

[CR11] Bert L, Parrot S, Robert F, Desvignes C, Denoroy L, Suaud-Chagny MF, Renaud B (2002). In vivo temporal sequence of rat striatal glutamate, aspartate and dopamine efflux during apomorphine, nomifensine, NMDA and PDC in situ administration. Neuropharmacology.

[CR12] Bianchi L, Colivicchi MA, Bolam JP, Della Corte L (1998). The release of amino acids from rat neostriatum and substantia nigra in vivo: a dual microdialysis probe analysis. Neuroscience.

[CR13] Biggs CS, Starr MS (1997). Dopamine and glutamate control each other's release in the basal ganglia: a microdialysis study of the entopeduncular nucleus and substantia nigra. Neurosci Biobehav Rev.

[CR14] Biggs CS, Pearce BR, Fowler LJ, Whitton PS (1992). The effect of sodium valproate on extracellular GABA and other amino acids in the rat ventral hippocampus: an in vivo microdialysis study. Brain Res.

[CR15] Biggs CS, Fowler LJ, Whitton PS, Starr MS (1995). Impulse-dependent and tetrodotoxin-sensitive release of GABA in the rat's substantia nigra measured by microdialysis. Brain Res.

[CR16] Biggs CS, Fowler LJ, Whitton PS, Starr MS (1997). Extracellular levels of glutamate and aspartate in the entopeduncular nucleus of the rat determined by microdialysis: regulation by striatal dopamine D2 receptors via the indirect striatal output pathway?. Brain Res.

[CR17] Boulet S, Lacombe E, Carcenac C, Feuerstein C, Sgambato-Faure V, Poupard A, Savasta M (2006). Subthalamic stimulation-induced forelimb dyskinesias are linked to an increase in glutamate levels in the substantia nigra pars reticulata. J Neurosci.

[CR18] Bourdelais AJ, Deutch AY (1994). The effects of haloperidol and clozapine on extracellular GABA levels in the prefrontal cortex of the rat: an in vivo microdialysis study. Cereb Cortex.

[CR19] Brand I, Fliegel S, Spanagel R, Noori HR (2013). Global ethanol-induced enhancements of monoaminergic neurotransmission: A meta-analysis study. Alcohol Clin Exp Res.

[CR20] Bustamante D, You ZB, Castel MN, Johansson S, Goiny M, Terenius L, Hokfelt T, Herrera-Marschitz M (2002). Effect of single and repeated methamphetamine treatment on neurotransmitter release in substantia nigra and neostriatum of the rat. J Neurochem.

[CR21] Calcagno E, Carli M, Invernizzi RW (2006). The 5-HT(1A) receptor agonist 8-OH-DPAT prevents prefrontocortical glutamate and serotonin release in response to blockade of cortical NMDA receptors. J Neurochem.

[CR22] Carboni S, Isola R, Gessa GL, Rossetti ZL (1993). Ethanol prevents the glutamate release induced by N-methyl-D-aspartate in the rat striatum. Neurosci Lett.

[CR23] Carli M, Calcagno E, Mainolfi P, Mainini E, Invernizzi RW (2011). Effects of aripiprazole, olanzapine, and haloperidol in a model of cognitive deficit of schizophrenia in rats: relationship with glutamate release in the medial prefrontal cortex. Psychopharmacology (Berl).

[CR24] Castaneda TR, de Prado BM, Prieto D, Mora F (2004). Circadian rhythms of dopamine, glutamate and GABA in the striatum and nucleus accumbens of the awake rat: modulation by light. J Pineal Res.

[CR25] Chapman MA, See RE (1996). Differential effects of unique profile antipsychotic drugs on extracellular amino acids in the ventral pallidum and globus pallidus of rats. J Pharmacol Exp Ther.

[CR26] Clinckers R, Gheuens S, Smolders I, Meurs A, Ebinger G, Michotte Y (2005). In vivo modulatory action of extracellular glutamate on the anticonvulsant effects of hippocampal dopamine and serotonin. Epilepsia.

[CR27] Cowen M, Chen F, Jarrott B, Lawrence AJ (1998). Effects of acute ethanol on GABA release and GABA(A) receptor density in the rat mesolimbic system. Pharmacol Biochem Behav.

[CR28] Cui WY, Seneviratne C, Gu J, Li MD (2012). Genetics of GABAergic signaling in nicotine and alcohol dependence. Hum Genet.

[CR29] Dahchour A, De Witte P (1999). Effect of repeated ethanol withdrawal on glutamate microdialysate in the hippocampus. Alcohol Clin Exp Res.

[CR30] Dahchour A, De Witte P (1999). Acamprosate decreases the hypermotility during repeated ethanol withdrawal. Alcohol.

[CR31] Dahchour A, De Witte P (2000). Taurine blocks the glutamate increase in the nucleus accumbens microdialysate of ethanol-dependent rats. Pharmacol Biochem Behav.

[CR32] Dahchour A, De Witte P (2003). Excitatory and inhibitory amino acid changes during repeated episodes of ethanol withdrawal: an in vivo microdialysis study. Eur J Pharmacol.

[CR33] Dahchour A, Quertemont E, De Witte P (1994). Acute ethanol increases taurine but neither glutamate nor GABA in the nucleus accumbens of male rats: a microdialysis study. Alcohol Alcohol.

[CR34] Dahchour A, Quertemont E, De Witte P (1996). Taurine increases in the nucleus accumbens microdialysate after acute ethanol administration to naive and chronically alcoholised rats. Brain Res.

[CR35] Dahchour A, De Witte P, Bolo N, Nedelec JF, Muzet M, Durbin P, Macher JP (1998). Central effects of acamprosate: part 1. Acamprosate blocks the glutamate increase in the nucleus accumbens microdialysate in ethanol withdrawn rats. Psychiatr Res.

[CR36] Dahchour A, Hoffman A, Deitrich R, de Witte P (2000). Effects of ethanol on extracellular amino acid levels in high-and low-alcohol sensitive rats: a microdialysis study. Alcohol Alcohol.

[CR37] Dalby NO (2000). GABA-level increasing and anticonvulsant effects of three different GABA uptake inhibitors. Neuropharmacology.

[CR38] Dalley JW, Thomas KL, Howes SR, Tsai TH, Aparicio-Legarza MI, Reynolds GP, Everitt BJ, Robbins TW (1999). Effects of excitotoxic lesions of the rat prefrontal cortex on CREB regulation and presynaptic markers of dopamine and amino acid function in the nucleus accumbens. Eur J Neurosci.

[CR39] Dawson LA, Nguyen HQ, Li P (2001). The 5-HT(6) receptor antagonist SB-271046 selectively enhances excitatory neurotransmission in the rat frontal cortex and hippocampus. Neuropsychopharmacology.

[CR40] Dawson LA, Nguyen HQ, Li P (2003). Potentiation of amphetamine-induced changes in dopamine and 5-HT by a 5-HT(6) receptor antagonist. Brain Res Bull.

[CR41] de Groote L, Linthorst AC (2007). Exposure to novelty and forced swimming evoke stressor-dependent changes in extracellular GABA in the rat hippocampus. Neuroscience.

[CR42] Del Arco A, Mora F (1999). Effects of endogenous glutamate on extracellular concentrations of GABA, dopamine, and dopamine metabolites in the prefrontal cortex of the freely moving rat: involvement of NMDA and AMPA/KA receptors. Neurochem Res.

[CR43] Del Arco A, Mora F (2000). Endogenous dopamine potentiates the effects of glutamate on extracellular GABA in the prefrontal cortex of the freely moving rat. Brain Res Bull.

[CR44] Del Arco A, Mora F (2002). NMDA and AMPA/kainate glutamatergic agonists increase the extracellular concentrations of GABA in the prefrontal cortex of the freely moving rat: modulation by endogenous dopamine. Brain Res Bull.

[CR45] Del Arco A, Castaneda TR, Mora F (1998). Amphetamine releases GABA in striatum of the freely moving rat: involvement of calcium and high affinity transporter mechanisms. Neuropharmacology.

[CR46] Ding ZM, Rodd ZA, Engleman EA, Bailey JA, Lahiri DK, McBride WJ (2012). Alcohol drinking and deprivation alter basal extracellular glutamate concentrations and clearance in the mesolimbic system of alcohol-preferring (P) rats. Addict Biol.

[CR47] Domart MC, Benyamina A, Lemoine A, Bourgain C, Blecha L, Debuire B, Reynaud M, Saffroy R (2012). Association between a polymorphism in the promoter of a glutamate receptor subunit gene (GRIN2A) and alcoholism. Addict Biol.

[CR48] Dong HL, Fukuda S, Murata E, Higuchi T (2006). Excitatory and inhibitory actions of isoflurane on the cholinergic ascending arousal system of the rat. Anesthesiology.

[CR49] Ericson M, Chau P, Clarke RB, Adermark L, Söderpalm B (2011). Rising taurine and ethanol concentrations in nucleus accumbens interact to produce dopamine release after ethanol administration. Addict Biol.

[CR50] Fantin M, Marti M, Auberson YP, Morari M (2007). NR2A and NR2B subunit containing NMDA receptors differentially regulate striatal output pathways. J Neurochem.

[CR51] Feng YZ, Zhang T, Rockhold RW, Ho IK (1995). Increased locus coeruleus glutamate levels are associated with naloxone-precipitated withdrawal from butorphanol in the rat. Neurochem Res.

[CR52] Feng Y, Rockhold RW, Ho IK (1997). Nor-binaltorphimine precipitates withdrawal and excitatory amino acid release in the locus ceruleus of butorphanol–but not morphine-dependent rats. J Pharmacol Exp Ther.

[CR53] Ferraro L, O'Connor WT, Li XM, Rimondini R, Beani L, Ungerstedt U, Fuxe K, Tanganelli S (1996). Evidence for a differential cholecystokinin-B and -A receptor regulation of GABA release in the rat nucleus accumbens mediated via dopaminergic and cholinergic mechanisms. Neuroscience.

[CR54] Ferraro L, Tanganelli S, O'Connor WT, Antonelli T, Rambert F, Fuxe K (1996). The vigilance promoting drug modafinil decreases GABA release in the medial preoptic area and in the posterior hypothalamus of the awake rat: possible involvement of the serotonergic 5-HT3 receptor. Neurosci Lett.

[CR55] Ferraro L, Antonelli T, O'Connor WT, Tanganelli S, Rambert F, Fuxe K (1997). The antinarcoleptic drug modafinil increases glutamate release in thalamic areas and hippocampus. Neuroreport.

[CR56] Ferraro L, O'Connor WT, Antonelli T, Fuxe K, Tanganelli S (1997). Differential effects of intrastriatal neurotensin(1–13) and neurotensin(8–13) on striatal dopamine and pallidal GABA release. A dual-probe microdialysis study in the awake rat. Eur J Neurosci.

[CR57] Ferraro L, Antonelli T, O'Connor WT, Tanganelli S, Rambert FA, Fuxe K (1998). The effects of modafinil on striatal, pallidal and nigral GABA and glutamate release in the conscious rat: evidence for a preferential inhibition of striato-pallidal GABA transmission. Neurosci Lett.

[CR58] Ferraro L, Antonelli T, Tanganelli S, O'Connor WT, Perez de la Mora M, Mendez-Franco J, Rambert FA, Fuxe K (1999). The vigilance promoting drug modafinil increases extracellular glutamate levels in the medial preoptic area and the posterior hypothalamus of the conscious rat: prevention by local GABAA receptor blockade. Neuropsychopharmacology.

[CR59] Ferraro L, O'Connor WT, Glennon J, Tomasini MC, Bebe BW, Tanganelli S, Antonelli T (2000). Evidence for a nucleus accumbens CCK2 receptor regulation of rat ventral pallidal GABA levels: a dual probe microdialysis study. Life Sci.

[CR60] Ferraro L, Tomasini MC, Fernandez M, Bebe BW, O'Connor WT, Fuxe K, Glennon JC, Tanganelli S, Antonelli T (2001). Nigral neurotensin receptor regulation of nigral glutamate and nigroventral thalamic GABA transmission: a dual-probe microdialysis study in intact conscious rat brain. Neuroscience.

[CR61] Forray MI, Bustos G, Gysling K (1999). Noradrenaline inhibits glutamate release in the rat bed nucleus of the stria terminalis: in vivo microdialysis studies. J Neurosci Res.

[CR62] Frank ST, Krumm B, Spanagel R (2008). Cocaine-induced dopamine overflow within the nucleus accumbens measured by in vivo microdialysis: a meta-analysis. Synapse.

[CR63] Frank J, Cichon S, Treutlein J, Ridinger M, Mattheisen M, Hoffmann P, Herms S, Wodarz N, Soyka M, Zill P, Maier W, Mössner R, Gaebel W, Dahmen N, Scherbaum N, Schmäl C, Steffens M, Lucae S, Ising M, Müller-Myhsok B, Nöthen MM, Mann K, Kiefer F, Rietschel M (2012). Genome-wide significant association between alcohol dependence and a variant in the ADH gene cluster. Addict Biol.

[CR64] Frantz K, Harte M, Ungerstedt U, WT OC (2002). A dual probe characterization of dialysate amino acid levels in the medial prefrontal cortex and ventral tegmental area of the awake freely moving rat. J Neurosci Meth.

[CR65] Fu Y, Matta SG, Gao W, Brower VG, Sharp BM (2000). Systemic nicotine stimulates dopamine release in nucleus accumbens: re-evaluation of the role of N-methyl-D-aspartate receptors in the ventral tegmental area. J Pharmacol Exp Ther.

[CR66] Galeffi F, Bianchi L, Bolam JP, Della Corte L (2003). The effect of 6-hydroxydopamine lesions on the release of amino acids in the direct and indirect pathways of the basal ganglia: a dual microdialysis probe analysis. Eur J Neurosci.

[CR67] Gass JT, Olive MF (2008). Glutamatergic substrates of drug addiction and alcoholism. Biochem Pharmacol.

[CR68] Gass JT, Sinclair CM, Cleva RM, Widholm JJ, Olive MF (2011). Alcohol-seeking behavior is associated with increased glutamate transmission in basolateral amygdala and nucleus accumbens as measured by glutamate-oxidase-coated biosensors. Addict Biol.

[CR69] Giorgetti M, Hotsenpiller G, Ward P, Teppen T, Wolf ME (2001). Amphetamine-induced plasticity of AMPA receptors in the ventral tegmental area: effects on extracellular levels of dopamine and glutamate in freely moving rats. J Neurosci.

[CR70] Giovannini MG, Mutolo D, Bianchi L, Michelassi A, Pepeu G (1994). NMDA receptor antagonists decrease GABA outflow from the septum and increase acetylcholine outflow from the hippocampus: a microdialysis study. J Neurosci.

[CR71] Giovannini MG, Rakovska A, Della Corte L, Bianchi L, Pepeu G (1998). Activation of non-NMDA receptors stimulates acetylcholine and GABA release from dorsal hippocampus: a microdialysis study in the rat. Neurosci Lett.

[CR72] Giovannini MG, Rakovska A, Benton RS, Pazzagli M, Bianchi L, Pepeu G (2001). Effects of novelty and habituation on acetylcholine, GABA, and glutamate release from the frontal cortex and hippocampus of freely moving rats. Neuroscience.

[CR73] Giovannini MG, Pazzagli M, Malmberg-Aiello P, Della Corte L, Rakovska AD, Cerbai F, Casamenti F, Pepeu G (2005). Inhibition of acetylcholine-induced activation of extracellular regulated protein kinase prevents the encoding of an inhibitory avoidance response in the rat. Neuroscience.

[CR74] Glass GV (1976). Primary, secondary and meta-analysis of research. Educ Res.

[CR75] Grobin AC, Deutch AY (1998). Dopaminergic regulation of extracellular gamma-aminobutyric acid levels in the prefrontal cortex of the rat. J Pharmacol Exp Ther.

[CR76] Guevara-Guzman R, Barrera-Mera B, De La Riva C, Kendrick KM (2000). Release of classical transmitters and nitric oxide in the rat olfactory bulb, evoked by vaginocervical stimulation and potassium, varies with the oestrus cycle. Eur J Neurosci.

[CR77] Guzman-Ramos K, Osorio-Gomez D, Moreno-Castilla P, Bermudez-Rattoni F (2010). Off-line concomitant release of dopamine and glutamate involvement in taste memory consolidation. J Neurochem.

[CR78] Hampp G, Ripperger JA, Houben T, Schmutz I, Blex C, Perreau-Lenz S, Brunk I, Spanagel R, Ahnert-Hilger G, Meijer JH, Albrecht U (2008). Regulation of monoamine oxidase A by circadian-clock components implies clock influence on mood. Curr Biol.

[CR79] Harte M, O'Connor WT (2004). Evidence for a differential medial prefrontal dopamine D1 and D2 receptor regulation of local and ventral tegmental glutamate and GABA release: a dual probe microdialysis study in the awake rat. Brain Res.

[CR80] Harte M, O'Connor WT (2005). Evidence for a selective prefrontal cortical GABA(B) receptor-mediated inhibition of glutamate release in the ventral tegmental area: a dual probe microdialysis study in the awake rat. Neuroscience.

[CR81] Hascup ER, Hascup KN, Stephens M, Pomerleau F, Huettl P, Gratton A, Gerhardt GA (2010). Rapid microelectrode measurements and the origin and regulation of extracellular glutamate in rat prefrontal cortex. J Neurochem.

[CR82] Hashimoto A, Oka T, Nishikawa T (1995). Extracellular concentration of endogenous free D-serine in the rat brain as revealed by in vivo microdialysis. Neuroscience.

[CR83] Hatzipetros T, Yamamoto BK (2006). Dopaminergic and GABAergic modulation of glutamate release from rat subthalamic nucleus efferents to the substantia nigra. Brain Res.

[CR84] Hazell AS, Butterworth RF, Hakim AM (1993). Cerebral vulnerability is associated with selective increase in extracellular glutamate concentration in experimental thiamine deficiency. J Neurochem.

[CR85] Hedges LV, Olkin I (1985). Statistical methods for meta-analysis, Chapter 8.

[CR86] Hemmati P, Shilliam CS, Hughes ZA, Shah AJ, Roberts JC, Atkins AR, Hunter AJ, Heidbreder CA (2001). In vivo characterization of basal amino acid levels in subregions of the rat nucleus accumbens: effect of a dopamine D(3)/D(2) agonist. Neurochem Int.

[CR87] Hermann D, Weber-Fahr W, Sartorius A, Hoerst M, Frischknecht U, Tunc-Skarka N, Perreau-Lenz S, Hansson AC, Krumm B, Kiefer F, Spanagel R, Mann K, Ende G, Sommer WH (2012). Translational magnetic resonance spectroscopy reveals excessive central glutamate levels during alcohol withdrawal in humans and rats. Biol Psychiatr.

[CR88] Hernandez LF, Segovia G, Mora F (2003). Effects of activation of NMDA and AMPA glutamate receptors on the extracellular concentrations of dopamine, acetylcholine, and GABA in striatum of the awake rat: a microdialysis study. Neurochem Res.

[CR89] Hernandez LF, Segovia G, Mora F (2008). Chronic treatment with a dopamine uptake blocker changes dopamine and acetylcholine but not glutamate and GABA concentrations in prefrontal cortex, striatum and nucleus accumbens of the awake rat. Neurochem Int.

[CR90] Herrera-Marschitz M, You ZB, Goiny M, Meana JJ, Silveira R, Godukhin OV, Chen Y, Espinoza S, Pettersson E, Loidl CF, Lubec G, Andersson K, Nylander I, Terenius L, Ungerstedt U (1996). On the origin of extracellular glutamate levels monitored in the basal ganglia of the rat by in vivo microdialysis. J Neurochem.

[CR91] Hondo H, Nakahara T, Nakamura K, Hirano M, Uchimura H, Tashiro N (1995). The effect of phencyclidine on the basal and high potassium evoked extracellular GABA levels in the striatum of freely-moving rats: an in vivo microdialysis study. Brain Res.

[CR92] Hoshi K, Ma T, Ho IK (1996). Precipitated kappa-opioid receptor agonist withdrawal increase glutamate in rat locus coeruleus. Eur J Pharmacol.

[CR93] Hoshi K, Ma T, Oh S, Ho IK (1997). Increased release of excitatory amino acids in rat locus coeruleus in kappa-opioid agonist dependent rats precipitated by nor-binaltorphimine. Brain Res.

[CR94] Hossain MM, Suzuki T, Unno T, Komori S, Kobayashi H (2008). Differential presynaptic actions of pyrethroid insecticides on glutamatergic and GABAergic neurons in the hippocampus. Toxicology.

[CR95] Hotsenpiller G, Wolf ME (2003). Baclofen attenuates conditioned locomotion to cues associated with cocaine administration and stabilizes extracellular glutamate levels in rat nucleus accumbens. Neuroscience.

[CR96] Huang M, Li Z, Dai J, Shahid M, Wong EH, Meltzer HY (2008). Asenapine increases dopamine, norepinephrine, and acetylcholine efflux in the rat medial prefrontal cortex and hippocampus. Neuropsychopharmacology.

[CR97] Hugues S, Garcia R, Lena I (2007). Time course of extracellular catecholamine and glutamate levels in the rat medial prefrontal cortex during and after extinction of conditioned fear. Synapse.

[CR98] Inui T, Yamamoto T, Shimura T (2009). GABAergic transmission in the rat ventral pallidum mediates a saccharin palatability shift in conditioned taste aversion. Eur J Neurosci.

[CR99] Invernizzi RW, Pierucci M, Calcagno E, Di Giovanni G, Di Matteo V, Benigno A, Esposito E (2007). Selective activation of 5-HT(2C) receptors stimulates GABA-ergic function in the rat substantia nigra pars reticulata: a combined in vivo electrophysiological and neurochemical study. Neuroscience.

[CR100] Ito K, Abekawa T, Koyama T (2006). Valproate blocks high-dose methamphetamine-induced behavioral cross-sensitization to locomotion-inducing effect of dizocilpine (MK-801), but not methamphetamine. Psychopharmacology (Berl).

[CR101] Juhasz G, Kekesi KA, Nyitrai G, Dobolyi A, Krogsgaard-Larsen P, Schousboe A (1997). Differential effects of nipecotic acid and 4,5,6,7-tetrahydroisoxazolo[4,5-c]pyridin-3-ol on extracellular gamma-aminobutyrate levels in rat thalamus. Eur J Pharmacol.

[CR102] Kalivas PW (2009). The glutamate homeostasis hypothesis of addiction. Nat Rev Neurosci.

[CR103] Karpyak VM, Geske JR, Colby CL, Mrazek DA, Biernacka JM (2012). Genetic variability in the NMDA-dependent AMPA trafficking cascade is associated with alcohol dependence. Addict Biol.

[CR104] Kashkin VA, De Witte P (2004). Ethanol but not acetaldehyde induced changes in brain taurine: a microdialysis study. Amino Acids.

[CR105] Katoh H, Sima K, Nawashiro H, Wada K, Chigasaki H (1997). The effect of MK-801 on extracellular neuroactive amino acids in hippocampus after closed head injury followed by hypoxia in rats. Brain Res.

[CR106] Kaura S, Bradford HF, Young AM, Croucher MJ, Hughes PD (1995). Effect of amygdaloid kindling on the content and release of amino acids from the amygdaloid complex: in vivo and in vitro studies. J Neurochem.

[CR107] Keck ME, Sillaber I, Ebner K, Welt T, Toschi N, Kaehler ST, Singewald N, Philippu A, Elbel GK, Wotjak CT, Holsboer F, Landgraf R, Engelmann M (2000). Acute transcranial magnetic stimulation of frontal brain regions selectively modulates the release of vasopressin, biogenic amines and amino acids in the rat brain. Eur J Neurosci.

[CR108] König JFKKR (1974). The rat brain.

[CR109] Koob GF (2004). A role for GABA mechanisms in the motivational effects of alcohol. Biochem Pharmacol.

[CR110] Kretschmer BD, Goiny M, Herrera-Marschitz M (2000). Effect of intracerebral administration of NMDA and AMPA on dopamine and glutamate release in the ventral pallidum and on motor behavior. J Neurochem.

[CR111] Kuntz A, Clement HW, Lehnert W, van Calker D, Hennighausen K, Gerlach M, Schulz E (2004). Effects of secretin on extracellular amino acid concentrations in rat hippocampus. J Neural Transm.

[CR112] Lallemand F, Ward RJ, Dravolina O, De Witte P (2006). Nicotine-induced changes of glutamate and arginine in naive and chronically alcoholized rats: an in vivo microdialysis study. Brain Res.

[CR113] Langlais PJ, Zhang SX (1993). Extracellular glutamate is increased in thalamus during thiamine deficiency-induced lesions and is blocked by MK-801. J Neurochem.

[CR114] Li X, Li J, Gardner EL, Xi ZX (2010). Activation of mGluR7s inhibits cocaine-induced reinstatement of drug-seeking behavior by a nucleus accumbens glutamate-mGluR2/3 mechanism in rats. J Neurochem.

[CR115] Li Z, Boules M, Williams K, Peris J, Richelson E (2010). The novel neurotensin analog NT69L blocks phencyclidine (PCP)-induced increases in locomotor activity and PCP-induced increases in monoamine and amino acids levels in the medial prefrontal cortex. Brain Res.

[CR116] Lillrank SM, O'Connor WT, Oja SS, Ungerstedt U (1994). Systemic phencyclidine administration is associated with increased dopamine, GABA, and 5-HIAA levels in the dorsolateral striatum of conscious rats: an in vivo microdialysis study. J Neural Transm Gen Sect.

[CR117] Lindefors N, Hurd YL, O'Connor WT, Brene S, Persson H, Ungerstedt U (1992). Amphetamine regulation of acetylcholine and gamma-aminobutyric acid in nucleus accumbens. Neuroscience.

[CR118] Littlewood CL, Jones N, O'Neill MJ, Mitchell SN, Tricklebank M, Williams SC (2006). Mapping the central effects of ketamine in the rat using pharmacological MRI. Psychopharmacology (Berl).

[CR119] Liu N, Ho IK, Rockhold RW (1999). Contribution of glutamatergic systems in locus coeruleus to nucleus paragigantocellularis stimulation-evoked behavior. Pharmacol Biochem Behav.

[CR120] Lovinger DM, White G, Weight FF (1989). Ethanol inhibits NMDA-activated ion current in hippocampal neurons. Science.

[CR121] Lupinsky D, Moquin L, Gratton A (2010). Interhemispheric regulation of the medial prefrontal cortical glutamate stress response in rats. J Neurosci.

[CR122] Mark KA, Soghomonian JJ, Yamamoto BK (2004). High-dose methamphetamine acutely activates the striatonigral pathway to increase striatal glutamate and mediate long-term dopamine toxicity. J Neurosci.

[CR123] Marti M, Guerrini R, Beani L, Bianchi C, Morari M (2002). Nociceptin/orphanin FQ receptors modulate glutamate extracellular levels in the substantia nigra pars reticulata. A microdialysis study in the awake freely moving rat. Neuroscience.

[CR124] Mason PA, Escarciga R, Doyle JM, Romano WF, Berger RE, Donnellan JP (1997). Amino acid concentrations in hypothalamic and caudate nuclei during microwave-induced thermal stress: analysis by microdialysis. Bioelectromagnetics.

[CR125] Massieu L, Morales-Villagran A, Tapia R (1995). Accumulation of extracellular glutamate by inhibition of its uptake is not sufficient for inducing neuronal damage: an in vivo microdialysis study. J Neurochem.

[CR126] Matuszewich L, Yamamoto BK (1999). Modulation of GABA release by dopamine in the substantia nigra. Synapse.

[CR127] Meeusen R, Smolders I, Sarre S, de Meirleir K, Keizer H, Serneels M, Ebinger G, Michotte Y (1997). Endurance training effects on neurotransmitter release in rat striatum: an in vivo microdialysis study. Acta Physiol Scand.

[CR128] Melani A, Pantoni L, Bordoni F, Gianfriddo M, Bianchi L, Vannucchi MG, Bertorelli R, Monopoli A, Pedata F (2003). The selective A2A receptor antagonist SCH 58261 reduces striatal transmitter outflow, turning behavior and ischemic brain damage induced by permanent focal ischemia in the rat. Brain Res.

[CR129] Melendez RI, Hicks MP, Cagle SS, Kalivas PW (2005). Ethanol exposure decreases glutamate uptake in the nucleus accumbens. Alcohol Clin Exp Res.

[CR130] Melis MR, Succu S, Mascia MS, Cortis L, Argiolas A (2004). Extracellular excitatory amino acids increase in the paraventricular nucleus of male rats during sexual activity: main role of N-methyl-d-aspartic acid receptors in erectile function. Eur J Neurosci.

[CR131] Mihic SJ, Ye Q, Wick MJ, Koltchine VV, Krasowski MD, Finn SE, Mascia MP, Valenzuela CF, Hanson KK, Greenblatt EP, Harris RA, Harrison NL (1997). Sites of alcohol and volatile anaesthetic action on GABA(A) and glycine receptors. Nature.

[CR132] Mikhailova MO (2003). Comparison of changes in glutamate levels in the nucleus accumbens of the rat brain during food consumption in conditions of blockade of dopamine D1 and D2 receptors. Neurosci Behav Physiol.

[CR133] Molchanova S, Koobi P, Oja SS, Saransaari P (2004). Interstitial concentrations of amino acids in the rat striatum during global forebrain ischemia and potassium-evoked spreading depression. Neurochem Res.

[CR134] Molchanova S, Oja SS, Saransaari P (2004). Characteristics of basal taurine release in the rat striatum measured by microdialysis. Amino Acids.

[CR135] Morales-Villagran A, Tapia R (1996). Preferential stimulation of glutamate release by 4-aminopyridine in rat striatum in vivo. Neurochem Int.

[CR136] Morari M, O'Connor WT, Ungerstedt U, Fuxe K (1993). N-methyl-D-aspartic acid differentially regulates extracellular dopamine, GABA, and glutamate levels in the dorsolateral neostriatum of the halothane-anesthetized rat: an in vivo microdialysis study. J Neurochem.

[CR137] Morari M, O'Connor WT, Ungerstedt U, Fuxe K (1994). Dopamine D1 and D2 receptor antagonism differentially modulates stimulation of striatal neurotransmitter levels by N-methyl-D-aspartic acid. Eur J Pharmacol.

[CR138] Morari M, O'Connor WT, Ungerstedt U, Bianchi C, Fuxe K (1996). Functional neuroanatomy of the nigrostriatal and striatonigral pathways as studied with dual probe microdialysis in the awake rat–II. Evidence for striatal N-methyl-D-aspartate receptor regulation of striatonigral GABAergic transmission and motor function. Neuroscience.

[CR139] Morari M, Sbrenna S, Marti M, O'Connor WT, Bianchi C, Fuxe K, Beani L (1998). Evidence for a striatal NMDA receptor modulation of nigral glutamate release. A dual probe microdialysis study in the awake freely moving rat. Eur J Neurosci.

[CR140] Mucignat-Caretta C, Colivicchi MA, Fattori M, Ballini C, Bianchi L, Gabai G, Cavaggioni A, Della Corte L (2006). Species-specific chemosignals evoke delayed excitation of the vomeronasal amygdala in freely-moving female rats. J Neurochem.

[CR141] Noori HR (2012). The effects of the acute administration of low-dosage ethanol on the phasic neurochemical oscillations of the basal ganglia. Math Med Biol.

[CR142] Noori HR, Spanagel R, Hansson AC (2012). Neurocircuitry of modeling drug effects. Addict Biol.

[CR143] Noori HR, Fliegel S, Brand I, Spanagel R (2012). The impact of Acetylcholinesterase Inhibitors on the Extracellular Acetylcholine Concentrations in the Adult Rat Brain: A Meta-Analysis. Synapse.

[CR144] Northrop NA, Smith LP, Yamamoto BK, Eyerman DJ (2011). Regulation of glutamate release by alpha7 nicotinic receptors: differential role in methamphetamine-induced damage to dopaminergic and serotonergic terminals. J Pharmacol Exp Ther.

[CR145] Nyitrai G, Szarics E, Kovacs I, Kekesi KA, Juhasz G, Kardos J (1999). Effect of CGP 36742 on the extracellular level of neurotransmitter amino acids in the thalamus. Neurochem Int.

[CR146] Ochi M, Shiozaki S, Kase H (2004). Adenosine A(2A) receptor-mediated modulation of GABA and glutamate release in the output regions of the basal ganglia in a rodent model of Parkinson's disease. Neuroscience.

[CR147] O'Connor WT, Osborne PG, Ungerstedt U (1998). Tolerance to catalepsy following chronic haloperidol is not associated with changes in GABA release in the globus pallidus. Brain Res.

[CR148] O'Dell LE, Parsons LH (2004). Serotonin1B receptors in the ventral tegmental area modulate cocaine-induced increases in nucleus accumbens dopamine levels. J Pharmacol Exp Ther.

[CR149] Ohoyama K, Yamamura S, Hamaguchi T, Nakagawa M, Motomura E, Shiroyama T, Tanii H, Okada M (2011). Effect of novel atypical antipsychotic, blonanserin, on extracellular neurotransmitter level in rat prefrontal cortex. Eur J Pharmacol.

[CR150] Oreiro-Garcia MT, Vazquez-Illanes MD, Sierra-Paredes G, Sierra-Marcuno G (2007). Changes in extracellular amino acid concentrations in the rat hippocampus after in vivo actin depolymerization with latrunculin A. Neurochem Int.

[CR151] Parrot S, Bert L, Renaud B, Denoroy L (2003). Glutamate and aspartate do not exhibit the same changes in their extracellular concentrations in the rat striatum after N-methyl-D-aspartate local administration. J Neurosci Res.

[CR152] Paxinos G, Watson C (2007). The rat brain in stereotaxic coordinates.

[CR153] Pehek EA, Nocjar C, Roth BL, Byrd TA, Mabrouk OS (2006). Evidence for the preferential involvement of 5-HT2A serotonin receptors in stress- and drug-induced dopamine release in the rat medial prefrontal cortex. Neuropsychopharmacology.

[CR154] Pellegrino LKPA, Cushman AJ (1979). A stereotaxic atlas of the rat brain.

[CR155] Petkova-Kirova P, Rakovska A, Della Corte L, Zaekova G, Radomirov R, Mayer A (2008). Neurotensin modulation of acetylcholine, GABA, and aspartate release from rat prefrontal cortex studied in vivo with microdialysis. Brain Res Bull.

[CR156] Pistis M, Ferraro L, Pira L, Flore G, Tanganelli S, Gessa GL, Devoto P (2002). Delta(9)-tetrahydrocannabinol decreases extracellular GABA and increases extracellular glutamate and dopamine levels in the rat prefrontal cortex: an in vivo microdialysis study. Brain Res.

[CR157] Qi J, Han WY, Yang JY, Wang LH, Dong YX, Wang F, Song M, Wu CF (2012). Oxytocin regulates changes of extracellular glutamate and GABA levels induced by methamphetamine in the mouse brain. Addict Biol.

[CR158] Quarta D, Ferre S, Solinas M, You ZB, Hockemeyer J, Popoli P, Goldberg SR (2004). Opposite modulatory roles for adenosine A1 and A2A receptors on glutamate and dopamine release in the shell of the nucleus accumbens. Effects of chronic caffeine exposure. J Neurochem.

[CR159] Quertemont E, de Neuville J, De Witte P (1998). Changes in the amygdala amino acid microdialysate after conditioning with a cue associated with ethanol. Psychopharmacology (Berl).

[CR160] Quertemont E, Dahchour A, Ward RJ, Witte P (1999). Ethanol induces taurine release in the amygdala: an in vivo microdialysis study. Addict Biol.

[CR161] Quertemont E, Lallemand F, Colombo G, De Witte P (2000). Taurine and ethanol preference: a microdialysis study using Sardinian alcohol-preferring and non-preferring rats. Eur Neuropsychopharmacol.

[CR162] Rakovska A, Giovannini MG, Della Corte L, Kalfin R, Bianchi L, Pepeu G (1998). Neurotensin modulation of acetylcholine and GABA release from the rat hippocampus: an in vivo microdialysis study. Neurochem Int.

[CR163] Rea K, Lang Y, Finn DP (2009). Alterations in extracellular levels of gamma-aminobutyric acid in the rat basolateral amygdala and periaqueductal gray during conditioned fear, persistent pain and fear-conditioned analgesia. J Pain.

[CR164] Rewal M, Donahue R, Gill TM, Nie H, Ron D, Janak PH (2012). Alpha4 subunit-containing GABAA receptors in the accumbens shell contribute to the reinforcing effects of alcohol. Addict Biol.

[CR165] Reynolds NC, Lin W, Cameron CM, Roerig DL (1999). Extracellular perfusion of rat brain nuclei using microdialysis: a method for studying differential neurotransmitter release in response to neurotoxins. Brain Res Brain Res Protoc.

[CR166] Rimondini R, O'Connor WT, Ferre S, Sillard R, Agerberth B, Mutt V, Ungerstedt U, Fuxe K (1994). PEC-60 increases dopamine but not GABA release in the dorsolateral neostriatum of the halothane anaesthetized rat. An in vivo microdialysis study. Neurosci Lett.

[CR167] Rimondini R, O'Connor WT, Sillard R, Mutt V, Ungerstedt U, Fuxe K (1996). The secretory trypsin inhibitor like-peptide, PEC-60 increases dopamine D2 receptor agonist induced inhibition of GABA release in the dorsolateral neostriatum of the awake freely moving rat. An in vivo microdialysis study. Regul Pept.

[CR168] Robelet S, Melon C, Guillet B, Salin P, Kerkerian-Le Goff L (2004). Chronic L-DOPA treatment increases extracellular glutamate levels and GLT1 expression in the basal ganglia in a rat model of Parkinson's disease. Eur J Neurosci.

[CR169] Robert F, Bert L, Lambas-Senas L, Denoroy L, Renaud B (1996). In vivo monitoring of extracellular noradrenaline and glutamate from rat brain cortex with 2-min microdialysis sampling using capillary electrophoresis with laser-induced fluorescence detection. J Neurosci Meth.

[CR170] Roberto M, Madamba SG, Stouffer DG, Parsons LH, Siggins GR (2004). Increased GABA release in the central amygdala of ethanol-dependent rats. J Neurosci.

[CR171] Roberto M, Schweitzer P, Madamba SG, Stouffer DG, Parsons LH, Siggins GR (2004). Acute and chronic ethanol alter glutamatergic transmission in rat central amygdala: an in vitro and in vivo analysis. J Neurosci.

[CR172] Roberto M, Cruz MT, Gilpin NW, Sabino V, Schweitzer P, Bajo M, Cottone P, Madamba SG, Stouffer DG, Zorrilla EP, Koob GF, Siggins GR, Parsons LH (2010). Corticotropin releasing factor-induced amygdala gamma-aminobutyric Acid release plays a key role in alcohol dependence. Biol Psychiatr.

[CR173] Rosales MG, Martinez-Fong D, Morales R, Nunez A, Flores G, Gongora-Alfaro JL, Floran B, Aceves J (1997). Reciprocal interaction between glutamate and dopamine in the pars reticulata of the rat substantia nigra: a microdialysis study. Neuroscience.

[CR174] Rosi S, Giovannini MG, Lestage PJ, Munoz C, Corte LD, Pepeu G (2004). S 18986, a positive modulator of AMPA receptors with cognition-enhancing properties, increases ACh release in the hippocampus of young and aged rat. Neurosci Lett.

[CR175] Rossetti ZL, Carboni S (1995). Ethanol withdrawal is associated with increased extracellular glutamate in the rat striatum. Eur J Pharmacol.

[CR176] Rowley HL, Marsden CA, Martin KF (1995). Differential effects of phenytoin and sodium valproate on seizure-induced changes in gamma-aminobutyric acid and glutamate release in vivo. Eur J Pharmacol.

[CR177] Rozza A, Masoero E, Favalli L, Lanza E, Govoni S, Rizzo V, Montalbetti L (2000). Influence of different anaesthetics on extracellular aminoacids in rat brain. J Neurosci Meth.

[CR178] Saellstroem Baum S, Huebner A, Krimphove M, Morgenstern R, Badawy AA, Spies CD (2006). Nicotine stimulation on extracellular glutamate levels in the nucleus accumbens of ethanol-withdrawn rats in vivo. Alcohol Clin Exp Res.

[CR179] Sato M, Ago Y, Koda K, Nakamura S, Kawasaki T, Baba A, Matsuda T (2007). Role of postsynaptic serotonin1A receptors in risperidone-induced increase in acetylcholine release in rat prefrontal cortex. Eur J Pharmacol.

[CR180] Saulskaya NB, Mikhailova MO (2002). Feeding-induced decrease in extracellular glutamate level in the rat nucleus accumbens: dependence on glutamate uptake. Neuroscience.

[CR181] Saul'skaya NB, Mikhailova MO (2005). Vesicular and non-vesicular glutamate release in the nucleus accumbens in conditions of a forced change of behavioral strategy. Neurosci Behav Physiol.

[CR182] Saulskaya NB, Soloviova NA (2004). Tetrodotoxin-dependent glutamate release in the rat nucleus accumbens during concurrent presentation of appetitive and conditioned aversive stimuli. J Neurosci Meth.

[CR183] Sayin U, Timmerman W, Westerink BH (1995). The significance of extracellular GABA in the substantia nigra of the rat during seizures and anticonvulsant treatments. Brain Res.

[CR184] Schumann G, Johann M, Frank J, Preuss U, Dahmen N, Laucht M, Rietschel M, Rujescu D, Lourdusamy A, Clarke TK, Krause K, Dyer A, Depner M, Wellek S, Treutlein J, Szegedi A, Giegling I, Cichon S, Blomeyer D, Heinz A, Heath S, Lathrop M, Wodarz N, Soyka M, Spanagel R, Mann K (2008). Systematic analysis of glutamatergic neurotransmission genes in alcohol dependence and adolescent risky drinking behavior. Arch Gen Psychiatr.

[CR185] Segovia G, Del Arco A, Mora F (1997). Endogenous glutamate increases extracellular concentrations of dopamine, GABA, and taurine through NMDA and AMPA/kainate receptors in striatum of the freely moving rat: a microdialysis study. J Neurochem.

[CR186] Segovia G, Del Arco A, Mora F (1999). Effects of aging on the interaction between glutamate, dopamine, and GABA in striatum and nucleus accumbens of the awake rat. J Neurochem.

[CR187] Segovia G, Del Arco A, Prieto L, Mora F (2001). Glutamate-glutamine cycle and aging in striatum of the awake rat: effects of a glutamate transporter blocker. Neurochem Res.

[CR188] Selim M, Bradberry CW (1996). Effect of ethanol on extracellular 5-HT and glutamate in the nucleus accumbens and prefrontal cortex: comparison between the Lewis and Fischer 344 rat strains. Brain Res.

[CR189] Semba J, Sakai M, Miyoshi R, Kito S (1995). NG-monomethyl-L-arginine, an inhibitor of nitric oxide synthase, increases extracellular GABA in the striatum of the freely moving rat. Neuroreport.

[CR190] Shimizu K, Matsubara K, Uezono T, Kimura K, Shiono H (1998). Reduced dorsal hippocampal glutamate release significantly correlates with the spatial memory deficits produced by benzodiazepines and ethanol. Neuroscience.

[CR191] Shou M, Smith AD, Shackman JG, Peris J, Kennedy RT (2004). In vivo monitoring of amino acids by microdialysis sampling with on-line derivatization by naphthalene-2,3-dicarboxyaldehyde and rapid micellar electrokinetic capillary chromatography. J Neurosci Meth.

[CR192] Singewald N, Zhou GY, Schneider C (1995). Release of excitatory and inhibitory amino acids from the locus coeruleus of conscious rats by cardiovascular stimuli and various forms of acute stress. Brain Res.

[CR193] Sizemore GM, Co C, Smith JE (2000). Ventral pallidal extracellular fluid levels of dopamine, serotonin, gamma amino butyric acid, and glutamate during cocaine self-administration in rats. Psychopharmacology (Berl).

[CR194] Skorzewska A, Bidzinski A, Hamed A, Lehner M, Turzynska D, Sobolewska A, Szyndler J, Maciejak P, Wislowska-Stanek A, Plaznik A (2009). The effect of CRF and alpha-helical CRF((9–41)) on rat fear responses and amino acids release in the central nucleus of the amygdala. Neuropharmacology.

[CR195] Smith ML, Glass GV (1977). Meta-analysis of psychotherapy outcome studies. Am Psychol.

[CR196] Smith SE, Sharp T (1994). An investigation of the origin of extracellular GABA in rat nucleus accumbens measured in vivo by microdialysis. J Neural Transm Gen Sect.

[CR197] Smith A, Watson CJ, Frantz KJ, Eppler B, Kennedy RT, Peris J (2004). Differential increase in taurine levels by low-dose ethanol in the dorsal and ventral striatum revealed by microdialysis with on-line capillary electrophoresis. Alcohol Clin Exp Res.

[CR198] Sommer W, Rimondini R, O'Connor W, Hansson AC, Ungerstedt U, Fuxe K (1996). Intrastriatally injected c-fos antisense oligonucleotide interferes with striatonigral but not striatopallidal gamma-aminobutyric acid transmission in the conscious rat. Proc Natl Acad Sci USA.

[CR199] Sotomayor-Zarate R, Araya KA, Pereira P, Blanco E, Quiroz G, Pozo S, Carreno P, Andres ME, Forray MI, Gysling K (2010). Activation of GABA-B receptors induced by systemic amphetamine abolishes dopamine release in the rat lateral septum. J Neurochem.

[CR200] Spanagel R (2009). Alcoholism: a systems approach from molecular physiology to addictive behavior. Physiol Rev.

[CR201] Spanagel R, Kiefer F (2008). Drugs for Relapse Prevention of Alcoholism – 10 Years of Progress. Trends Pharmacol Sci.

[CR202] Spanagel R, Bartsch D, Brors B, Dahmen N, Deussing J, Eils R, Ende G, Gallinat J, Gebicke-Haerter P, Heinz A, Kiefer F, Jäger W, Mann K, Matthäus F, Nöthen M, Rietschel M, Sartorius A, Schütz G, Sommer WH, Sprengel R, Walter H, Wichmann E, Wienker T, Wurst W, Zimmer A (2010). An integrated genome research network for studying the genetics of alcohol addiction. Addict Biol.

[CR203] Stephans SE, Yamamoto BY (1995). Effect of repeated methamphetamine administrations on dopamine and glutamate efflux in rat prefrontal cortex. Brain Res.

[CR204] Succu S, Mascia MS, Sanna F, Melis T, Argiolas A, Melis MR (2006). The cannabinoid CB1 receptor antagonist SR 141716A induces penile erection by increasing extra-cellular glutamic acid in the paraventricular nucleus of male rats. Behav Brain Res.

[CR205] Sullivan ME, Hall SR, Milne B, Jhamandas K (2000). Suppression of acute and chronic opioid withdrawal by a selective soluble guanylyl cyclase inhibitor. Brain Res.

[CR206] Sun JY, Yang JY, Wang F, Wang JY, Song W, Su GY, Dong YX, Wu CF (2011). Lesions of nucleus accumbens affect morphine-induced release of ascorbic acid and GABA but not of glutamate in rats. Addict Biol.

[CR207] Takeda A, Sotogaku N, Oku N (2002). Manganese influences the levels of neurotransmitters in synapses in rat brain. Neuroscience.

[CR208] Takeda A, Sotogaku N, Oku N (2003). Influence of manganese on the release of neurotransmitters in rat striatum. Brain Res.

[CR209] Takeda A, Minami A, Seki Y, Oku N (2004). Differential effects of zinc on glutamatergic and GABAergic neurotransmitter systems in the hippocampus. J Neurosci Res.

[CR210] Tanaka Y, Han H, Hagishita T, Fukui F, Liu G, Ando S (2004). alpha-Sialylcholesterol enhances the depolarization-induced release of acetylcholine and glutamate in rat hippocampus: in vivo microdialysis study. Neurosci Lett.

[CR211] Tanganelli S, O'Connor WT, Ferraro L, Bianchi C, Beani L, Ungerstedt U, Fuxe K (1994). Facilitation of GABA release by neurotensin is associated with a reduction of dopamine release in rat nucleus accumbens. Neuroscience.

[CR212] Terzioglu B, Aypak C, Onat FY, Kucukibrahimoglu E, Ozkaynakci AE, Goren MZ (2006). The effects of ethosuximide on amino acids in genetic absence epilepsy rat model. J Pharmacol Sci.

[CR213] Timmerman W, Westerink BH (1997). Brain microdialysis of GABA and glutamate: what does it signify?. Synapse.

[CR214] Timmerman W, Cisci G, Nap A, de Vries JB, Westerink BH (1999). Effects of handling on extracellular levels of glutamate and other amino acids in various areas of the brain measured by microdialysis. Brain Res.

[CR215] Tokuyama S, Zhu H, Wakabayashi H, Feng YZ, Ho IK (1998). The role of glutamate in the locus coeruleus during opioid withdrawal and effects of H-7, a protein kinase inhibitor, on the action of glutamate in rats. J Biomed Sci.

[CR216] Toth E, Vizi ES, Lajtha A (1993). Effect of nicotine on levels of extracellular amino acids in regions of the rat brain in vivo. Neuropharmacology.

[CR217] Tsai GC, Coyle JT (1998). The role of glutamatergic neurotransmission in the pathophysiology of alcoholism. Ann Rev Med.

[CR218] Ueda Y, Tsuru N (1995). Simultaneous monitoring of the seizure-related changes in extracellular glutamate and gamma-aminobutyric acid concentration in bilateral hippocampi following development of amygdaloid kindling. Epilepsy Res.

[CR219] Uhart M, Weerts EM, McCaul ME, Guo X, Yan X, Kranzler HR, Li N, Wand GS (2012). GABRA2 markers moderate the subjective effects of alcohol. Addict Biol.

[CR220] Varga V, Kekesi A, Juhasz G, Kocsis B (1998). Reduction of the extracellular level of glutamate in the median raphe nucleus associated with hippocampal theta activity in the anaesthetized rat. Neuroscience.

[CR221] Vengeliene V, Bilbao A, Molander A, Spanagel R (2008). Neuropharmacology of alcohol addiction. Br J Pharmacol.

[CR222] Voisin DL, Chapman C, Poulain DA, Herbison AE (1994). Extracellular GABA concentrations in rat supraoptic nucleus during lactation and following haemodynamic changes: an in vivo microdialysis study. Neuroscience.

[CR223] Wang B, Shaham Y, Zitzman D, Azari S, Wise RA, You ZB (2005). Cocaine experience establishes control of midbrain glutamate and dopamine by corticotropin-releasing factor: a role in stress-induced relapse to drug seeking. J Neurosci.

[CR224] Wang L, Maher TJ, Wurtman RJ (2007). Oral L-glutamine increases GABA levels in striatal tissue and extracellular fluid. FASEB J.

[CR225] Welty N, Shoblock JR (2009). The effects of thioperamide on extracellular levels of glutamate and GABA in the rat prefrontal cortex. Psychopharmacology (Berl).

[CR226] Westphalen RI, Hemmings HC (2006). Volatile anesthetic effects on glutamate versus GABA release from isolated rat cortical nerve terminals: 4-aminopyridine-evoked release. J Pharmacol Exp Ther.

[CR227] Windels F, Bruet N, Poupard A, Urbain N, Chouvet G, Feuerstein C, Savasta M (2000). Effects of high frequency stimulation of subthalamic nucleus on extracellular glutamate and GABA in substantia nigra and globus pallidus in the normal rat. Eur J Neurosci.

[CR228] Windels F, Carcenac C, Poupard A, Savasta M (2005). Pallidal origin of GABA release within the substantia nigra pars reticulata during high-frequency stimulation of the subthalamic nucleus. J Neurosci.

[CR229] Winter C, Lemke C, Sohr R, Meissner W, Harnack D, Juckel G, Morgenstern R, Kupsch A (2008). High frequency stimulation of the subthalamic nucleus modulates neurotransmission in limbic brain regions of the rat. Exp Brain Res.

[CR230] Wislowska-Stanek A, Hamed A, Lehner M, Bidzinski A, Turzynska D, Sobolewska A, Walkowiak J, Plaznik A (2008). Effects of midazolam and buspirone on in vivo concentration of amino acids and monoamine metabolites in the rat hippocampus. Pharmacol Rep.

[CR231] Wolf ME, Xue CJ (1998). Amphetamine and D1 dopamine receptor agonists produce biphasic effects on glutamate efflux in rat ventral tegmental area: modification by repeated amphetamine administration. J Neurochem.

[CR232] Wolf ME, Xue CJ (1999). Amphetamine-induced glutamate efflux in the rat ventral tegmental area is prevented by MK-801, SCH 23390, and ibotenic acid lesions of the prefrontal cortex. J Neurochem.

[CR233] Xi ZX, Ramamoorthy S, Shen H, Lake R, Samuvel DJ, Kalivas PW (2003). GABA transmission in the nucleus accumbens is altered after withdrawal from repeated cocaine. J Neurosci.

[CR234] Xi ZX, Shen H, Baker DA, Kalivas PW (2003). Inhibition of non-vesicular glutamate release by group III metabotropic glutamate receptors in the nucleus accumbens. J Neurochem.

[CR235] Yamada T, Terashima T, Kawano S, Furuno R, Okubo T, Juneja LR, Yokogoshi H (2009). Theanine, gamma-glutamylethylamide, a unique amino acid in tea leaves, modulates neurotransmitter concentrations in the brain striatum interstitium in conscious rats. Amino Acids.

[CR236] Yamamoto Y, Kakigi T, Maeda K (1999). Intra-striatal phencyclidine inhibits N-methyl-D-aspartic acid-stimulated increase in glutamate levels of freely moving rats. Prog Neuropsychopharmacol B_ol. Psychiatry.

[CR237] Yamamura S, Ohoyama K, Hamaguchi T, Kashimoto K, Nakagawa M, Kanehara S, Suzuki D, Matsumoto T, Motomura E, Shiroyama T, Okada M (2009). Effects of quetiapine on monoamine, GABA, and glutamate release in rat prefrontal cortex. Psychopharmacology (Berl).

[CR238] Yamamura S, Ohoyama K, Nagase H, Okada M (2009). Zonisamide enhances delta receptor-associated neurotransmitter release in striato-pallidal pathway. Neuropharmacology.

[CR239] Yan QS, Reith ME, Yan SG, Jobe PC (1998). Effect of systemic ethanol on basal and stimulated glutamate releases in the nucleus accumbens of freely moving Sprague–Dawley rats: a microdialysis study. Neurosci Lett.

[CR240] Yan QS, Zheng SZ, Feng MJ, Yan SE (2005). Involvement of 5-HT1B receptors within the ventral tegmental area in ethanol-induced increases in mesolimbic dopaminergic transmission. Brain Res.

[CR241] Yoshida S, Okada M, Zhu G, Kaneko S (2007). Carbamazepine prevents breakdown of neurotransmitter release induced by hyperactivation of ryanodine receptor. Neuropharmacology.

[CR242] You ZB, Herrera-Marschitz M, Pettersson E, Nylander I, Goiny M, Shou HZ, Kehr J, Godukhin O, Hokfelt T, Terenius L, Ungerstedt U (1996). Modulation of neurotransmitter release by cholecystokinin in the neostriatum and substantia nigra of the rat: regional and receptor specificity. Neuroscience.

[CR243] You ZB, Saria A, Fischer-Colbrie R, Terenius L, Goiny M, Herrera-Marschitz M (1996). Effects of secretogranin II-derived peptides on the release of neurotransmitters monitored in the basal ganglia of the rat with in vivo microdialysis. Naunyn Schmiedebergs Arch Pharmacol.

[CR244] You ZB, Tzschentke TM, Brodin E, Wise RA (1998). Electrical stimulation of the prefrontal cortex increases cholecystokinin, glutamate, and dopamine release in the nucleus accumbens: an in vivo microdialysis study in freely moving rats. J Neurosci.

[CR245] You ZB, Chen YQ, Wise RA (2001). Dopamine and glutamate release in the nucleus accumbens and ventral tegmental area of rat following lateral hypothalamic self-stimulation. Neuroscience.

[CR246] You ZB, Wang B, Zitzman D, Azari S, Wise RA (2007). A role for conditioned ventral tegmental glutamate release in cocaine seeking. J Neurosci.

[CR247] Zangen A, Hyodo K (2002). Transcranial magnetic stimulation induces increases in extracellular levels of dopamine and glutamate in the nucleus accumbens. Neuroreport.

[CR248] Zhang T, Feng Y, Rockhold RW, Ho IK (1994). Naloxone-precipitated morphine withdrawal increases pontine glutamate levels in the rat. Life Sci.

[CR249] Zhu MY, Wang WP, Huang J, Feng YZ, Regunathan S, Bissette G (2008). Repeated immobilization stress alters rat hippocampal and prefrontal cortical morphology in parallel with endogenous agmatine and arginine decarboxylase levels. Neurochem Int.

[CR250] Zuiderwijk M, Veenstra E, Lopes da Silva FH, Ghijsen WE (1996). Effects of uptake carrier blockers SK & F 89976-A and L-trans-PDC on in vivo release of amino acids in rat hippocampus. Eur J Pharmacol.

